# A Cytosine‑Bulged (3+1) Hybrid G‑Quadruplex Formed by the Chicken DNA Replication Origin

**DOI:** 10.1002/chem.71389

**Published:** 2026-07-15

**Authors:** Yingying You, Monica Ching Suen, Haitao Miao, Yanyan Geng, Rahman Ud Din, Mu Yuan, Qiang Xue, Xiaoting Li, Naining Xu, Zhihong Guo, Hon‐Ming Lam, Kwok‐Wai Lo, Changdong Liu, Guang Zhu

**Affiliations:** ^1^ Department of Otorhinolaryngology Head and Neck Surgery, the Eighth Affiliated Hospital Sun Yat‐sen University Shenzhen China; ^2^ Division of Life Science The Hong Kong University of Science and Technology Hong Kong China; ^3^ Fujian Key Laboratory of Brain Tumors Diagnosis and Precision Treatment, Xiamen Key Laboratory of Brain Center, Clinical Research Institute of the First Affiliated Hospital of Xiamen University, the First Affiliated Hospital of Xiamen University, School of Medicine, School of Public Health Xiamen University Xiamen Fujian China; ^4^ HKUST Shenzhen Research Institute, Hi‐Tech Park Shenzhen China; ^5^ Department of Chemistry The Hong Kong University of Science and Technology Hong Kong China; ^6^ School of Life Sciences and the Centre for Soybean Research of the State Key Laboratory of Agrobiotechnology The Chinese University of Hong Kong Hong Kong China; ^7^ Department of Anatomical and Cellular Pathology, Prince of Wales Hospital, State Key Laboratory of Translational Oncology, Sir YK Pao Centre For Cancer The Chinese University of Hong Kong Hong Kong China; ^8^ Institute For Advanced Study and State Key Laboratory of Molecular Neuroscience The Hong Kong University of Science and Technology Hong Kong China

## Abstract

Guanine (G)‐rich nucleic acid sequences, capable of folding into G‐quadruplex (G4), are widely distributed in functionally important genomic regions and play key roles in DNA replication. Origin G‐rich repeated elements (OGRE) have been identified at replication origins in mouse, *Drosophila*, human and chicken. However, G4 structures formed by OGRE remain poorly understood. Here, we report the first high‑resolution G4 structure formed by OGRE from the β‐globin replication origin of chicken (*Gallus gallus*). The 23 nt DNA sequence d[GGGAACGCGGCATCAGGGTGGGG], termed *Ch*OGRE23, folds into an intramolecular three‐layer (3+1) hybrid G4 containing a cytosine bulge. Notably, the 5'‐end guanine participating in the outermost G‐quartet adopts an *anti* glycosidic conformation, representing a previously unobserved glycosidic alignment in unimolecular (3+1) hybrid G4s. Together with the cytosine bulge, these unusual structural features create a distinct molecular surface that may facilitate selective recognition by replication‑associated proteins or small molecules.

## Graphical abstract

The first high‐resolution structure of an OGRE‐derived G‐quadruplex from the chicken β‐globin replication origin reveals an intramolecular three‐layer (3+1) hybrid fold with a cytosine bulge and an unprecedented 5'‐outer‐quartet *anti* guanine, creating a distinctive surface for molecular recognition.

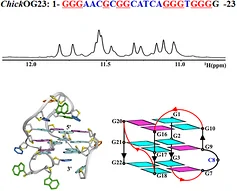

## 1 Introduction

DNA replication is an essential cellular process that is initiated at specific genomic sites known as replication origins. To maintain genome stability, each replication origin is tightly regulated to ensure that it fires no more than during a single cell cycle [1, 2, 3, 4]. In eukaryotic cells, assembly of the replication machinery is initiated by the origin recognition complex (ORC) binding to origin DNA, a process that requires ATP [5]. Notably, the sequence specificity of ORC binding varies significantly across species. In *Saccharomyces cerevisiae*, ORC binds with high sequence specificity to conserved autonomous replicating sequences (ARSs) in vitro [3, 6]. By contrast, ORC in *Schizosaccharomyces. pombe*, *Drosophila*, and human exhibits minimal sequence specificity and instead shows a preference for AT‐rich DNA [5, 7, 8]. Despite the conserved role of ORC in eukaryotic replication initiation, the universal DNA features that define replication origins and facilitate ORC recognition remain poorly understood.

Origin G‐rich Repeated Elements (OGREs), a class of Guanine (G)‐rich sequences, have been identified at the replication origins of diverse metazoans, including mouse, Drosophila, human, and chicken [8, 9, 10, 11, 12, 13, 14]. OGREs are characterized by their strong propensity to form G‐quadruplex (G4) structures [10, 11, 12, 14]. Importantly, several key proteins involved in DNA replication, including ORC, Mdm2‐binding protein (MTBP), and RecQL4, have been shown in vitro to exhibit binding preference for OGREs [15, 16, 17]. The functional significance of this interaction was first demonstrated by a study in chicken cells, where G4 formation was shown to be essential for replication initiation [18]. Subsequent studies in multiple systems, including mouse cells, avian DT40 cells, HEK293 cells and *Xenopus laevis* eggs, have further confirmed that G4 structures formed by OGREs play a critical role in DNA replication initiation in metazoans [19, 20]. Together, these studies suggest that replication‐associated proteins may recognize OGRE‐containing regions, potentially through the G4 structures formed by these sequences.

G‐rich nucleotide sequences can fold into G4 structures, which are stabilized by the stacking of G‐quartets. Each G‐quartet consists of four guanine bases arranged in a square planar geometry and connected through Hoogsteen hydrogen bonds [21]. The coordination of monovalent cations, particularly K^+^ which is the most physiologically relevant, is essential for G4 formation and stability [22]. Depending on strand orientation and loop connectivity, G4 structures can adopt diverse topologies, broadly classified as parallel, antiparallel, or hybrid (3+1) forms under physiological conditions [21, 23, 24]. Beyond these canonical architectures, increasing evidence supports the existence of structurally diverse G4 variants, including forms with bulges, interrupted G‐tracts, G‐triads, V‐loops, and interlocked or left‐handed G4s [25]. Importantly, G4 forming‐sequences harboring such features, particularly bulges, remain difficult to predict using current bioinformatic approaches or molecular dynamics simulations [26]. Therefore, elucidating the high‐resolution structures of these G4s is critical for understanding their roles in biological processes and for improving the prediction of G4s with specific structural features.

Despite extensive functional evidence implicating OGRE‑associated G4s in replication initiation, no high‑resolution structure of an OGRE G4 has been reported to date, limiting mechanistic understanding of protein recognition and origin regulation. To characterize the G4 structures formed by OGRE DNA sequences, we analyzed a 36‐nt DNA fragment from the β‐globin replication origin in chicken, d[GGGGGAGGGAACGCGGCATCAG GGTGGGGCTCAGGG] termed *Chick*OG36, using NMR spectroscopy. The NMR data revealed that *Chick*OG36 exists as a mixture of multiple G4 conformations, posing a significant challenge for structural determination (Figures 1 and S1). Consequently, a truncated derivative *Ch*OGRE23, d[GGGAACGCGGCATCAG GGTGGGG], was selected for further study as its well‐dispersed spectrum indicated a single predominant G4 conformation in solution (Figure 1). Here, we report the high‑resolution G4 structure of *Ch*OGRE23. Our findings reveal that *Ch*OGRE23 folds into a novel three‐layer (3+1) hybrid intramolecular G4 topology containing a cytosine bulge. Intriguingly, the first 5'‐end guanine involved in G‐quartet formation adopts an *anti* glycosidic conformation, a feature that is rarely observed in unimolecular (3+1) hybrid G4 structures.

**FIGURE 1:**
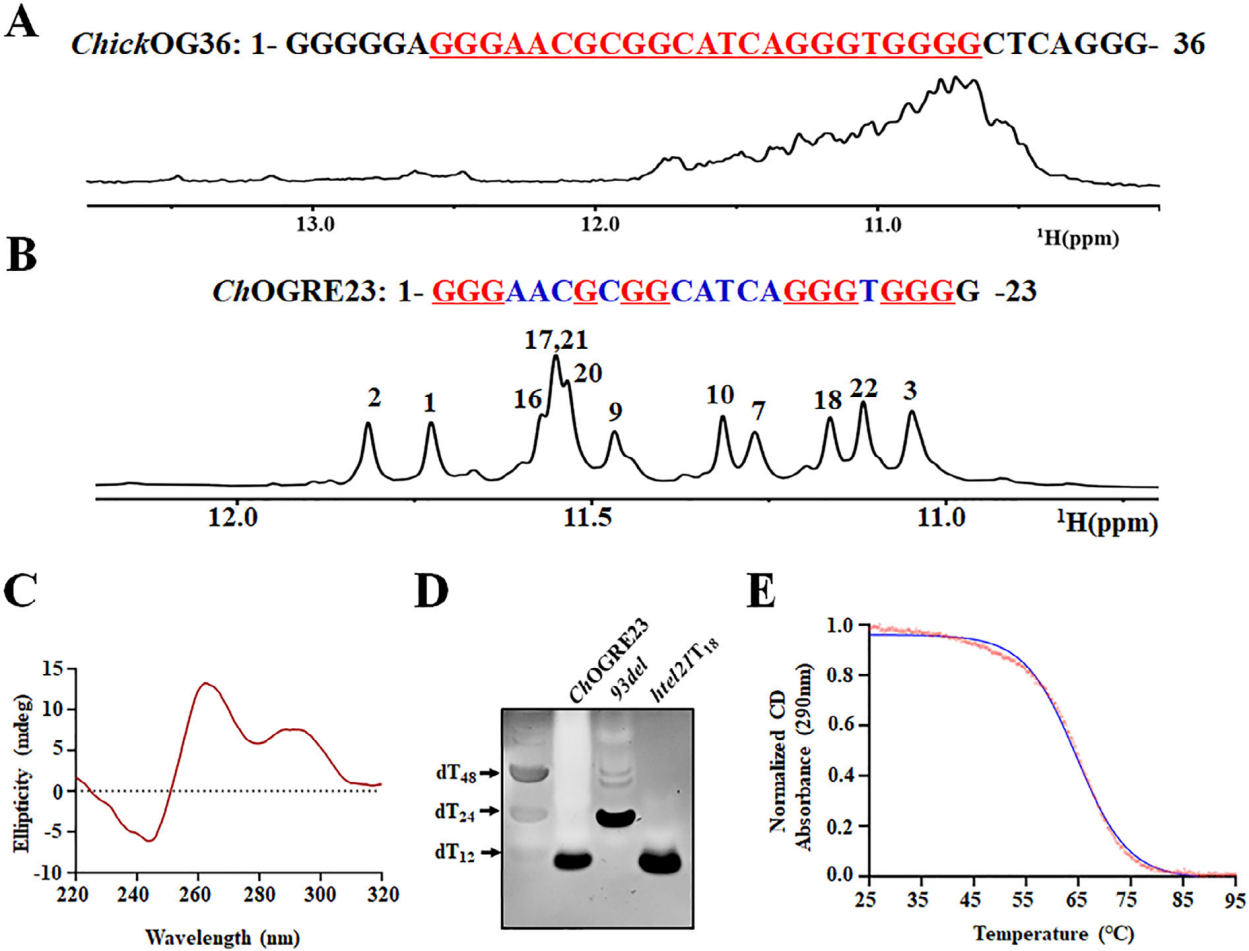
(A) DNA sequence of *Chick*OG36 and its imino proton region of the 1D ^1^H‐NMR spectrum. (B) DNA sequence of *Ch*OGRE23, with guanine residues involved in G‐quartet formation underlined, and the imino proton region of the 1D ^1^H‐NMR spectrum. Assignments of guanine imino resonances are indicated by nucleotide numbers on each resonance peak. Guanine imino protons were assigned using 1D ^15^N‐filtered HSQC spectra of samples containing site‐specific low‐enrichment (5%) ^15^N‐labelled oligonucleotides at the indicated positions. (C) Circular dichroism (CD) spectrum of *Ch*OGRE23 in 70 mM KCl and 20 mM potassium phosphate buffer collected recorded at 25°C. (D) Comparison of the native PAGE migration of of *Ch*OGRE23 with reference samples, DNA size markers (dT_12_, dT_24_ and dT_48_); *93del* (d[GGGGTGGGAGGAGGGT]), a dimeric G4; *htel21*T_18_, (d[(GGGTTA)_2_GGGTTTGGG]), a unimolecular G4. (E) The CD melting experiment was performed in 70 mM KCl and 20 mM potassium phosphate.

## 2 Material and Methods

### 2.1 Sample Preparation

Unlabeled nucleotides and site‐specific 5% ^15^N, ^13^C‐labeled low‐enrichment nucleotides were synthesized by BGI Genomics and Takara. All nucleotides were dissolved in annealing buffer containing 70 mM KCl and 20 mM potassium phosphate (pH 7.0) to a final concentration of 0.1 mM. Samples were annealed by heating to 95°C for 15 min, followed by gradual cooling to room temperature overnight.

For NMR experiments, annealed samples were exchanged into a buffer containing 70 mM KCl and 20 mM potassium phosphate (pH 7.0) prepared in either 10% or 100% D_2_O. Final DNA concentrations ranged from 0.1 to 1 mM. The DNA concentration was determined by measuring the UV absorbance at 260 nm.

### 2.2 Circular Dichroism (CD) Spectroscopy

CD spectra were collected at 25°C using an Applied Photophysics Chirascan CD spectrometer equipped with 1‐mm path‐length quartz cuvette and a sample volume of 400 µl. Samples were prepared at a strand concentration of 15 µM in 20 mM potassium phosphate buffer (pH 7.0) containing 70 mM KCl. Thermal melting experiments were performed by monitoring ellipticity at 290 nm over a temperature range of 25–95 °C with a heating rate of 1  °Cmin^−^^1^. Melting curves were fitted using a Boltzmann sigmoid function (Prism).

### 2.3 Polyacrylamide Gel Electrophoresis (PAGE)

Native PAGE was performed on a 25% polyacrylamide gel (acrylamide:bis‐acrylamide 29:1) containing 20 mM KCl. The running buffer comprised 0.5X TBE and 20 mM KCl. All DNA samples were prepared at a strand concentration of 100 µM, and the DNA bands were visualized by staining with RedSafe dye.

### 2.4 Nuclear Magnetic Resonance (NMR) Spectroscopy

NMR experiments were conducted on Varian INOVA 500 and 800 MHz spectrometers at 25°C. Site‐specific assignments of guanine imino protons were obtained using 1D ^15^N‐filtered HSQC experiments on 5% ^15^N, ^13^C site‐specifically labelled samples [27]. Guanine H8 protons were identified using either a 2D HMBC experiment at natural abundance or 2D ^13^C‐HSQC experiments on 5% ^15^N, ^13^C site‐specifically labelled DNA samples [28]. Standard 2D NMR experiments, including NOESY recorded with different mixing times, TOCSY and COSY, were collected at 25°C to achieve complete proton resonance assignments [24]. Water suppression was achieved using either Watergate or Jump‐and‐Return pulse sequences. The NMR data were processed using nmrPipe [29, 30], and peak assignment and integration were performed using the software Sparky (http://www.cgl.ucsf.edu/home/sparky/).

### 2.5 NMR Structural Calculation

Interproton distance restraints were derived from NOESY spectra recorded at mixing times of 50, 100, 150 and 250 ms in D_2_O solution and categorized based on peak intensities as strong (1.8 to 3.6 Å), medium (1.8 to 4.5 Å), weak (1.8 to 5.5 Å) or very weak (2.8 to 6.5 Å). Exchangeable proton restraints were obtained from NOESY experiments recorded in H_2_O solution at mixing times of 100, 150, 250 and 400 ms, categorized as strong (1.8 to 4.5 Å), medium (1.8 to 5.5 Å), weak (1.8 to 6.5 Å) and very weak (1.8 to 7.2 Å). Dihedral angle restraints were applied to constrain *syn*‐ and *anti*‐ glycosidic torsion angles (χ), 60(±35)° and 240(±40)°, respectively, based on experimental data [31, 32, 33]. All experimentally derived distance restraints, together with G‑quartet hydrogen‑bonding restraints, were included in the calculations.

Structural calculation of *Ch*OGRE23 was performed using XPLOR‐NIH [34] according to a previously described protocol [35]. An extended starting conformation of the oligonucleotide was used to generate initial folds by torsion angle dynamics guided by NMR restraints (Table S1). The system was heated for 60 ps at 40 000 K using a hybrid energy function comprising geometric and NOE terms, with the van der Waals (vdW) weight set to 0.1 and the NOE scaling factor set to 150. The structures were cooled from 40,000 to 1,000 K over 60 ps while the vdW weight was increased linearly from 0.1 to 1.0, followed by cooling from 1,000 to 300 K over 6 ps using Cartesian molecular dynamics [36]. Structures with no restraint violations and low energies were selected for further refinement using distance‐restrained molecular dynamics, which was initiated at 300 K, heated to 1 000 K over 6 ps, equilibrated at 1 000 K for 18 ps, cooled to 300 K over 14 ps, and equilibrated at 300 K for 12 ps. Coordinates saved every 0.5 ps during the final 4.0 ps were averaged and energy‑minimized until the gradient was <0.1 kcal·mol^−^^1^. A soft planarity restraint (10 kcal·mol^−^^1^·Å^−^^2^) was applied to G‑quartets prior to heating and removed at the start of equilibration. The 10 lowest‑energy structures without NOE violations were selected as the final ensemble. Refinement statistics are summarized in Table S1.

All molecular graphics were prepared using PyMOL (http://www.pymol.org).

## 3 Results

### 3.1 An Intramolecular (3+1) G4 Adopted by *Chogre23*

As shown in Figures 1A and S1, the ^1^H NMR spectrum of the 36‐nt *Chick*OG36 in the imino proton region exhibited a complex pattern, suggesting the coexistence of multiple G4 conformations. This observation is consistent with of the native PAGE result, in which *Chick*OG36 migrated as multiple bands. The circular dichroism (CD) spectrum of *Ch*i*ck*OG36 displayed a dominant positive peak at 265 nm, a negative peak at 240 nm and a weaker shoulder around 290 nm, indicative of a predominantly parallel G4 conformation with additional antiparallel strand contributions, possibly consistent with a hybrid topology. To identify a construct more suitable for high‐resolution structural analysis, we generated three truncated derivatives: *Ch*OGRE30, d[GGGAACGCGGCATCAGGGTGGGGCTCAGGG]; *Ch*OGRE29, d[GGGGGAGGGAACGCGGCATCAGGGTGGGG]; and *Ch*OGRE23, d[GGGAACGCGGCATCAG GGTGGGG] (Figure S1). *Ch*OGRE29 and *Ch*OGRE30 correspond to *Ch*OGRE23 with 5'‐ and 3'‐terminal extensions, respectively. The 1D ^1^H NMR spectrum of *Ch*OGRE23 displayed a relatively well‐resolved set of imino proton signals, consistent with a more homogeneous folded conformation. In contrast, *Ch*OGRE29 and *Ch*OGRE30 showed more complex imino proton patterns, indicative of conformational heterogeneity and the presence of multiple folded states in solution. Native PAGE analysis showed that *Ch*OGRE29 migrated as multiple bands, similar to *Chick*OG36, further supporting the presence of multiple conformations. Notably, the CD spectrum of *Ch*OGRE29 was also similar to that of *chick*OG36, suggesting that *Ch*OGRE29 retains key conformational features of the longer sequence. By contrast, *Ch*OGRE30 showed a major band in native PAGE, but its CD spectrum, characterized by a dominant positive peak at 290 nm, a negative peak at 240 nm and a weaker shoulder around 265 nm, also resembled that of *Chick*OG36 and was suggestive of a hybrid‐like G4 topology, consistent with the mixed conformational features observed for *Ch*OGRE23. Together, these results indicate that extension of the *Ch*OGRE23 core sequence by additional 5'‐ or 3'‐ nucleotides increases conformational heterogeneity, while preserving key structural features of the core fold in the longer sequence context. We therefore chose *Ch*OGRE23 as the minimal construct for detailed structural characterization by NMR.

The ^1^H NMR spectrum of *Ch*OGRE23 exhibited eleven resolved imino proton resonances between 11.0 and 12.0 ppm, indicating the formation of a major intramolecular G4 fold (Figure 1B). Subsequent site‐specific labeling experiments confirmed that these resonances correspond to twelve guanine imino protons, with one overlapped signal, consistent with the formation of a three‐layer G4 structure. In K^+^ solution, the CD spectrum of *Ch*OGRE2*3* displayed a dominant positive peak at 265 nm, a weaker secondary positive peak at 290 nm, and a negative peak at 240 nm, supporting a (3+1) hybrid G4 topology with mixed antiparallel and parallel strand orientations (Figure 1C) [37, 38, 39].

The guanine imino (H1) protons of *Ch*OGRE23 were unambiguously assigned by using a site‐specific low‐enrichment (5%) ^15^N‐labeling strategy (Figure S2) [27], revealing that 12 guanines participate in the formation of the G‐quartet core, whereas G23 at the 3'‐terminus does not. Notably, the native PAGE analysis demonstrated that *Ch*OGRE2*3* has electrophoretic mobility similar to that of *htel21*T_18_, a 21‐nt human telomeric variant repeat known to adopt a unimolecular chair‐type G4 [35], but greater than that of *del93*, a dimeric parallel G4 (Figure 1D) [40].

Altogether, these data indicate that *Ch*OGRE23 forms a three‐layered monomeric G4. Notably, *Ch*OGRE23 exhibits a relatively high thermal stability, with a *T_m_* of 64.6°C in 70 mM KCl and 20 mM phosphate buffer at pH 7.0, as determined by a CD melting experiment (Figure 1E).

### 3.2 NMR Resonance Assignment and Determination of Glycosidic Torsion Angle

The unambiguously assigned imino protons in the 1D ^1^H‐NMR spectra (Figure S2) enabled subsequent resonance assignments using 2D NMR experiments. A natural‐abundance 2D HMBC experiment was performed to establish correlations between guanine H8 and H1 protons within the same residue, thereby allowing assignment of the H8 resonances of G1, G10, G16‐G18 and G20‐G22 as shown in Figure 2A. The remaining guanine H8 resonances, together with H1' resonances of G2, G3, G7, G9 and G23, were clearly identified using 2D ^1^H‐^13^C HSQC spectra recorded on the low‐enrichment (5%) ^15^N, ^13^C site‐specific labelled oligonucleotides as shown in Figure 2B. The C8‐H8 cross‐peak of G1 was identified in the 2D HMBC spectrum (Figure 2A). However, the corresponding C8‐H8 cross‐peak of G1 was not observed in the 2D ^1^H‐^13^C HSQC spectrum of the 5% ^15^N, ^13^C site‐specific labelled sample. In addition, the C8‐H8 cross‐peak of G1 remained undetectable in the expanded aromatic C8‐H8/C6‐H6 region of the 2D ^1^H‐^13^C HSQC spectrum of *Ch*OGRE23 (Figure S3), possibly because of line broadening associated with local dynamics or exchange processes at the 5'‐terminus. Therefore, the G1 was assigned primarily based on H8/H1' NOE connectivities involving neighbouring residues in NOESY spectrum (Figure 3).

**FIGURE 2:**
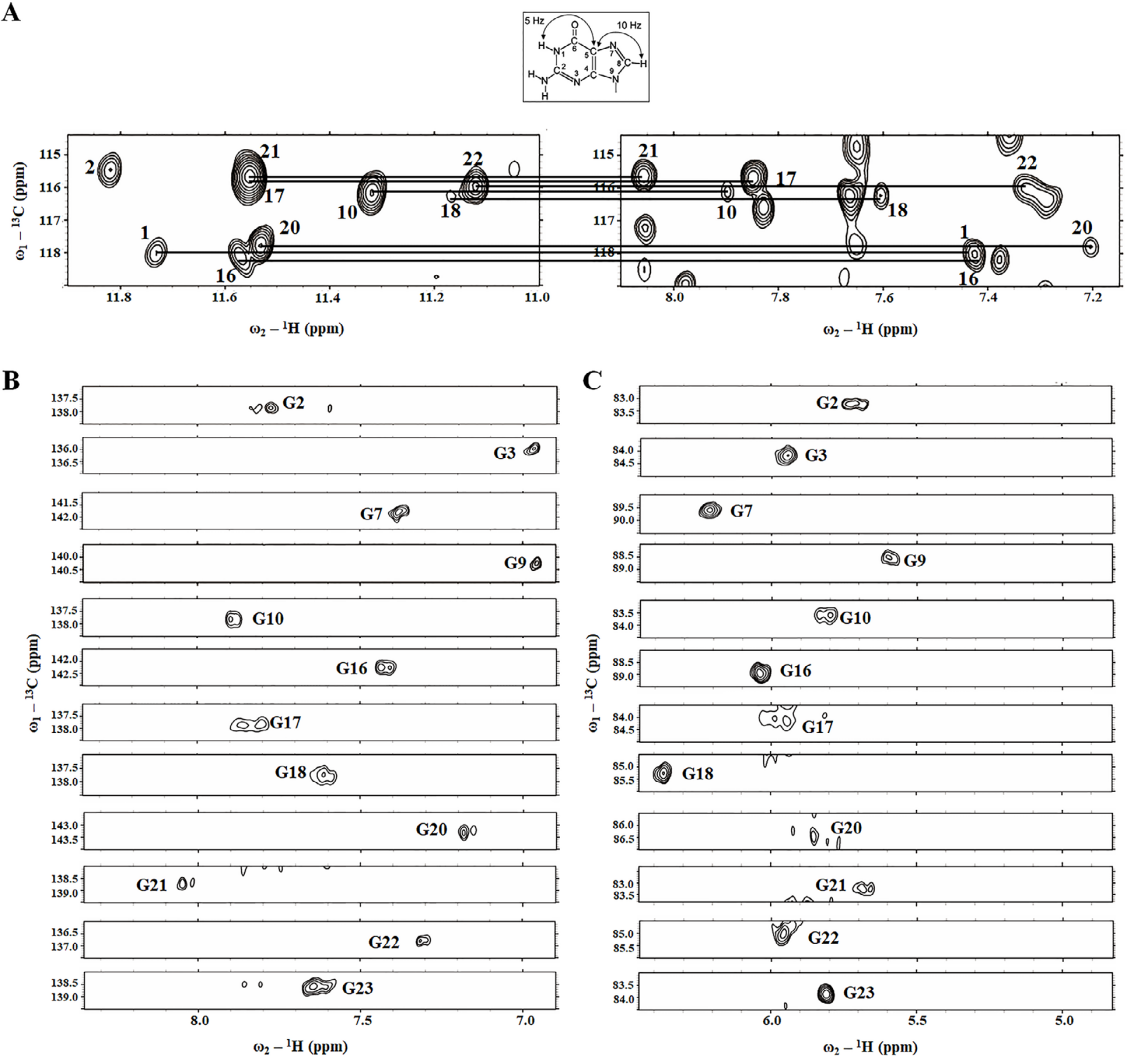
(A) Imino and H8 regions of 2D HMBC spectrum of *Ch*OGRE23, showing correlations between guanosine imino protons and H8 protons. *Top*: through‐bond correlations between imino and H8 protons via guanosine C5 carbon at natural abundance through long‐range J couplings. The spectrum was acquired at 800 MHz and 25°C in 70 mM KCl, 20 mM potassium phosphate buffer containing 10% D_2_O (pH 7.0). (B) Expanded H8‐C8 and (C) ribose H1'‐C1' regions of the ^13^C‐^1^H HSQC spectra of *Ch*OGRE23 guanosines. Samples contained site‐specifically low‐enrichment (5%) ^15^N/^13^C‐labelled oligonucleotides at positions 2, 3, 7, 9, 10, 16, 17, 18, 20, 21, 22 and 23.

**FIGURE 3:**
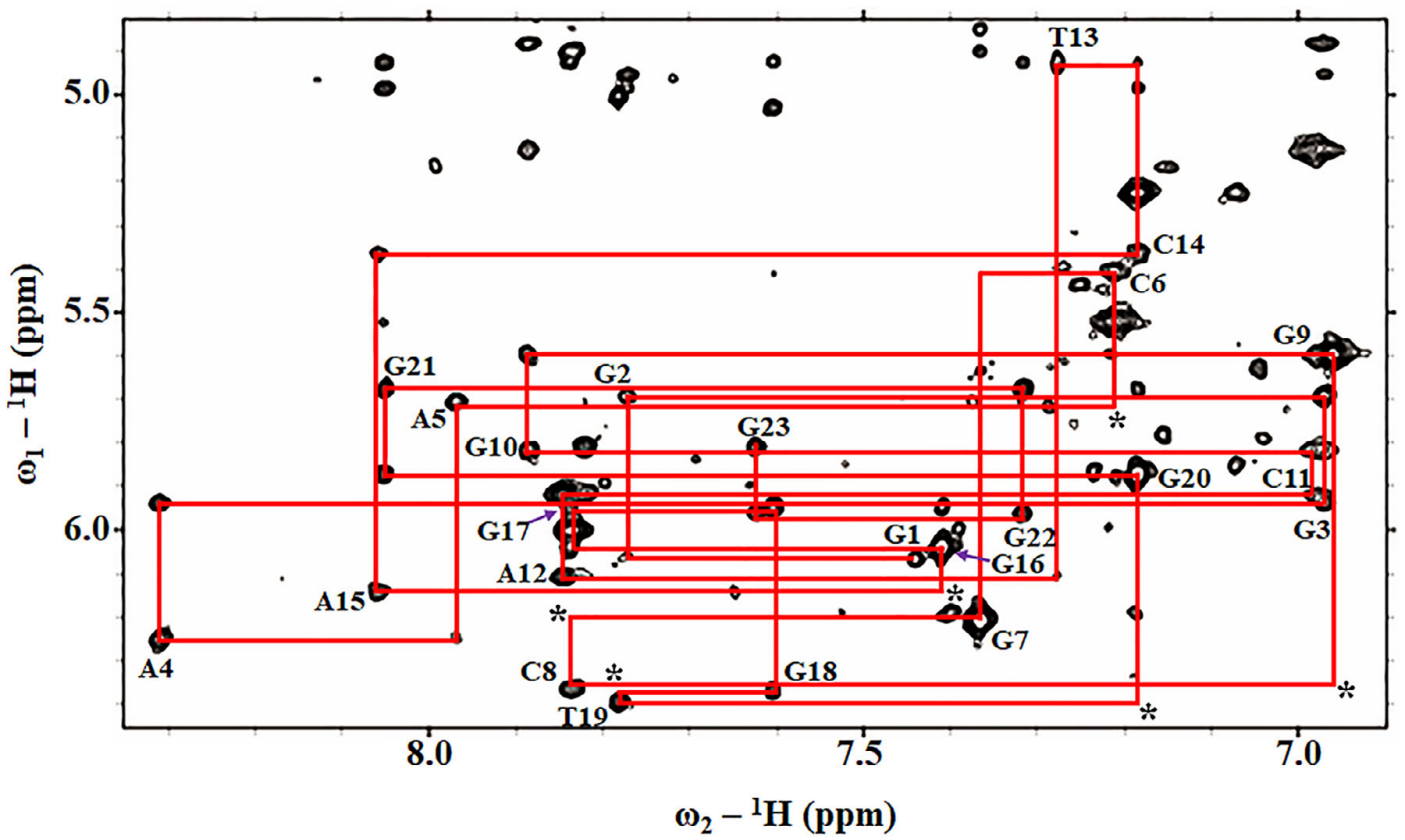
^1^H‐^1^H NOESY spectrum of *Ch*OGRE23 recorded in 100% D_2_O at 25°C with a mixing time of 250 ms, showing H6/H8‐H1' connectivities. The expanded region displays correlations between base H8 and sugar H1' protons. Four guanines, G7, G9, G16 and G20, adopt the *syn* conformation, as indicated by strong intra‐residue H1'‐H8 NOE cross‐peaks. *Anti*G(i)H1'/*anti*G(i+1)H8 NOEs were observed for *anti*G1‐*anti*G2, *anti*G2‐*anti*G3, *anti*G17‐*anti*G18, *anti*G21‐*anti*G22 and *anti*G22‐*anti*G23, whereas characteristic *syn*G(i)H1'/*anti*G(i+1)H8 and *syn*G(i)H8/*anti*G(i+1)H1' NOEs were detected for *syn*G9‐*anti*G10, *syn*G16‐*anti*G17 and *syn*G20*anti*‐G21 NOEs. Misissing G(i)H1'/G(i+1)H8 cross‐peaks, A5‐C6, G7‐C8 and A15‐G16, are marked by asterisks.

Assignments of H1' protons of all residues were achieved on the basis of 2D NOESY spectra by identifying intra‐residue H1'‐H6/H8 correlations. In Figure 3, the sequential H6/H8‐H1' connectivities were traced and further verified by the H2'/H2''‐H6/H8 NOE correlations. The H2' and H2'' protons were identified and stereospecifically assigned using a combination of 2D COSY, TOCSY, and NOESY spectra (Figures S4 and S5). Assignment of most H3', H4' and H5'/H5'' sugar protons was obtained from 2D NOESY spectra in combination with 2D COSY and TOCSY experiments. Cytosine residues were assigned on the basis of their characteristic H5‐H6 cross‐peaks via the 2D COSY and TOCSY experiments (Figure S6). These assignments were further confirmed by comparison with the 2D COSY spectra of the C‐to‐T substitution mutants C6T, C8T, and C11T (Figure S7). The T13 and T19 methyl groups were assigned based on their characteristic correlations in the 2D COSY spectrum and verified using the 2D COSY spectra of the T‐to‐C substitution mutant T19C (Figure S8). These assignments were further confirmed by the characteristic H6‐H1' NOE cross‐peaks in the 2D NOESY spectrum.

To further validate the sequential H6/H8–H1' correlations for A4, C6, C8, C11, A15, and T19, single‐site mutants with A‐to‐T, T‐to‐C or C‐to‐T substitutions were employed. Most mutants exhibited NMR spectra comparable to that of *Ch*OGRE23, indicating preservation of the same G4 fold. In contrast, A5T, A12T, T13C, and C14T showed heterogeneous spectra, indicative of conformational heterogeneity and/or alteration of the G4 fold (Figure S9).

In the 2D NOESY spectrum obtained with a mixing time of 50 ms, G7, G9, G16, and G20 exhibited intense intra‐residue H1'‐H8 cross‐peaks, a hallmark of the *syn* glycosidic conformation (Figure S10). By contrast, the other nine guanines in *Ch*OGRE23 adopt the *anti* glycosidic conformations. Although the C5 chemical shift of G1 appears closer to those of typical *syn* guanines than to those of typical *anti* guanines [41], this feature may reflect local terminal effects associated with its 5'‐ terminal position. Accordingly, G1 was assigned as adopting an *anti* conformation on the basis of the H8/H1' NOE intensity pattern in the short‐mixing‐time (50 ms) NOESY spectrum. Furthermore, the *syn*‐to‐*anti* step of G9‐G10, G16‐G17 and G20‐G21 displayed the distinctive rectangular NOE patterns defined by *syn*G(i)H1'/*anti*G(i+1)H8 and *syn*G(i)H8/*anti*G(i+1)H1' correlations, corroborating the glycosidic torsion angle assignments (Figure S11).

### 3.3 Determination of Folding Topology of *Ch*OGRE23, a Hybrid Unimolecular (3+1) G4 With a Cytosine‐Bulge

Unambiguous assignments of guanine imino (H1) and H8 protons in the 2D NOESY spectrum recorded with a 250 ms mixing time (Figure 4A) enabled direct determination of the folding topology of *Ch*OGRE2*3*. Within a Hoogsteen hydrogen‐bonded G‑quartet, each guanine imino H1 is in close spatial proximity to the H8 of an adjacent guanine (Figure 4B), giving rise to characteristic NOE signatures for G‐quartet identification.

**FIGURE 4:**
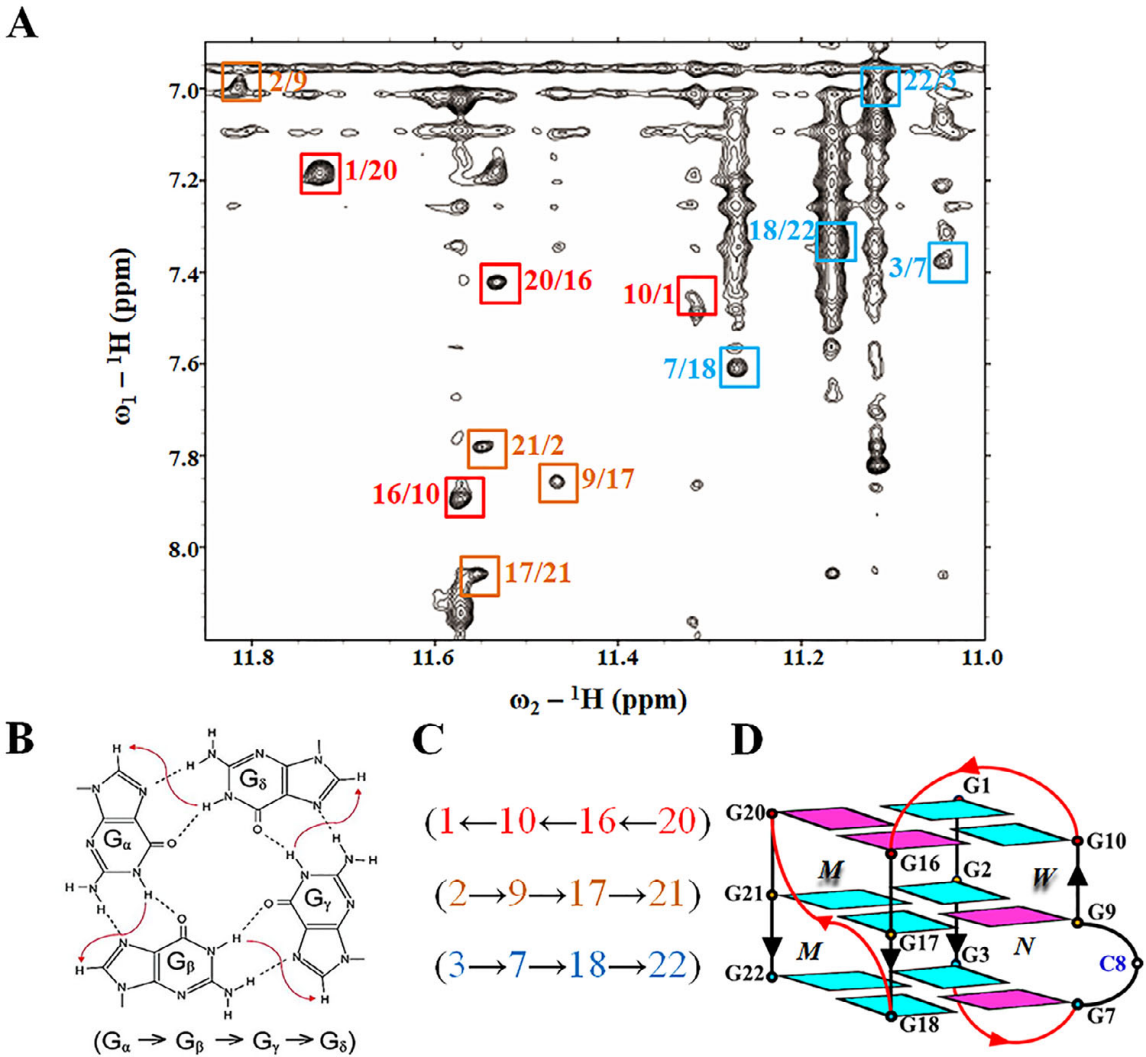
Folding topology of *Ch*OGRE23. (A) ^1^H‐^1^H NOESY spectrum of *Ch*OGRE23 recorded in 10% D_2_O at 25°C with a 250 ms mixing time, illustrating H1‐H8 connectivities. NOE cross‐peaks defining the three G‐quartets are boxed and labelled by the residue numbers of the H1 and H8 protons. (B) Characteristic guanine H1‐H8 NOE connectivity patterns in a Gα·Gβ·Gγ·Gδ G‐quartet. (C) Guanine H1‐H8 NOE connectivities observed for the three G‐quartets, G1·G10·G16·G21, G2·G9·G17·G21, and G3·G7·G18·G22, which are shown in red, orange, and blue, respectively. (D) Schematic depiction of the topology adopted by *Ch*OGRE2*3*, a hybrid (3+1) form. *Anti‐* and *syn*‐guanines are shown in cyan and magenta, respectively. The G4 core and loop segments are shown in black and red, respectively.

Analysis of H1‐H8 NOE cross‐peaks established the presence of three stacked G‐quartets formed by G1·G10·G16·G20, G2·G9·G17·G21 and G3·G7·G18·G22 (Figure 4C). Notably, the G2·G9·G17·G21 and G3·G7·G18·G22 G‐quartets display hydrogen‐bond directionality opposite to that of the G1·G10·G16·G20 G‐quartet (Figure 4C). Specifically, hydrogen bonding proceeds in an anticlockwise orientation in the G1·G10·G16·G20 G‐quartet, but in a clockwise orientation in the G2·G9·G17·G21 and G3·G7·G18·G22 G‐quartets. The glycosidic conformations within each G‐quartet were further supported by characteristic guanine H1–H1 and H8–H8 NOE cross‐peaks (Figure S12). The top G‐quartet adopts an *anti*·*anti*·*syn*·*syn* alignment, whereas the middle and bottom G‐quartets both exhibit an *anti*·*syn*·*anti*·*anti* alignment.

Accordingly, *Ch*OGRE2*3* folds into a three‐layer intramolecular (3 + 1) G4, in which three strands run in the same direction, whereas the fourth strand runs antiparallel to them (Figure 4D). In particular, the four strands are connected by two lateral loops and one propeller loop. Notably, the second G‐tract is interrupted by a cytosine residue at position 8, giving rise to an extruded bulge (Figure 4D).

### 3.4 The NMR Structure of *Ch*OGRE23 G4

The NMR structure of *Ch*OGRE23 in K^+^ solution was calculated on the basis of NOE restraints (Table S1) derived from NOESY spectra. The ensemble of the ten refined lowest‐energy conformers is depicted in Figure 5A. While the G4 core is well converged, the loop regions exhibit greater structural variability. A representative structure from the refined ensemble is shown in ribbon representation in Figure 5B.

**FIGURE 5:**
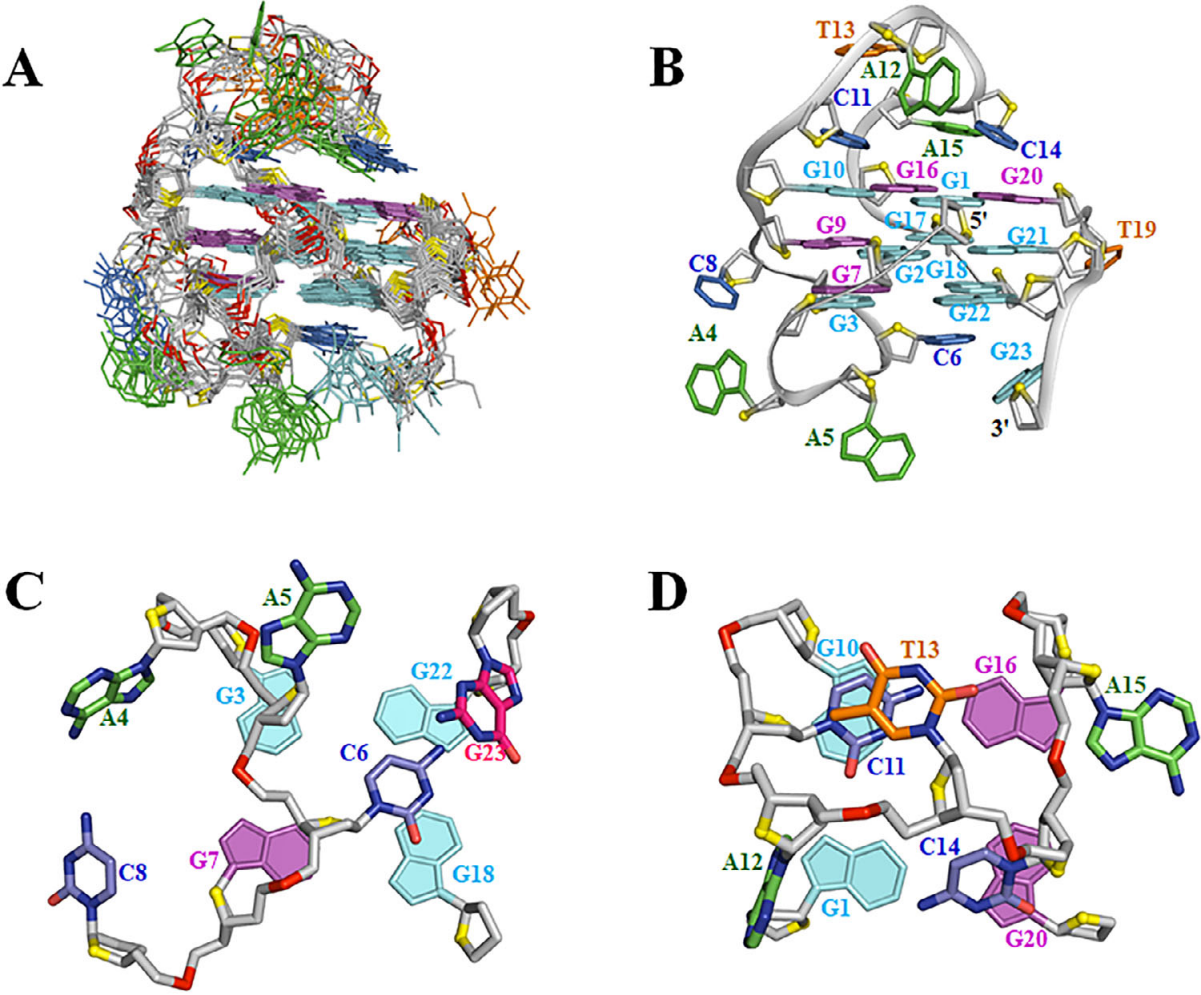
G4 structure of *Ch*OGRE23. (A) Superposition of the 10 lowest‑energy refined structures (PDB code: 9WNM). (B) Ribbon view of the lowest‐energy refined structure. Guanines adopting *anti‐* and *syn*‐glycosidic conformations are colored cyan and magenta, respectively; thymine, adenine, and cytosine residues are colored orange, green, and blue, respectively. The sugar–phosphate backbone is shown in gray, with O4' atoms highlighted in yellow and phosphorus atoms in red. Expanded views of the loop regions. (C, D) Expanded views of the loop (C) A4‐A5‐C6 loop conformation together with the C8 cytosine bulge. (D) Local conformation of the C11‐A12‐T13‐C14‐A15 segment from the representative structure with the lowest energy.

In *Ch*OGRE23, the strand arrangement defines a groove architecture composed of one wide, one narrow, and two medium grooves. Its A4‐A5‐C6 segment forms a lateral loop that links strands across the wide groove on one end of the G4 core, whereas the C11‐A12‐T13‐C14‐A15segment forms the second lateral loop over the narrow groove on the opposite end. The remaining loop, T19, is a single‐residue propeller loop spanning a medium groove between two parallel strands. According to the G4 reference framework established by Webba da Silva [42, 43], this combination of strand orientation and loop connectivity is classified as a (−l, −l, −p) topology.

The two lateral loops of *Ch*OGRE23 display distinct conformational features. At the bottom of the G4 core, A4 and A5 are solvent‐exposed and project away from the core in opposite directions, whereas C6 lies over the G3·G7·G18·G22 G‐quartet in an approximately coplanar orientation, capping the G‐quartet through stacking interactions (Figure 5C). This arrangement is supported by NOEs between the imino protons of G18 and G22 and the H5/H6 protons of C6. The upper lateral loop is more ordered at its flanking cytosines, C11 and C14, which stack onto guanine residues of the top G‐quartet, as supported by NOEs between the imino protons of G1, G16 and G20 and the H5/H6 protons of C11 and C14. Additional NOEs between the H8 proton of G10 and the H5/H6 protons of C11 further support the positioning of C11. Moreover, no imino proton signals were observed between 12.0‐14.0 ppm, indicating the absence of G∙C or A∙T Watson‐Crick base pairs in *Ch*OGRE23 (Figure S1). By contrast, the intervening residues A12, T13, and A15 are less well defined, indicating greater local mobility (Figure 5D). Substitutions at A12, T13, and C14 caused the largest perturbations in the imino proton region in the 1D ^1^H NMR spectrum, indicating important roles in maintaining the G4 fold (Figure S9). In contrast, mutations at other loop positions, including C6 and C11, produced comparatively smaller spectral changes, suggesting that these residues contribute to local stabilization but are less critical for the overall fold. This indicates that loop residues can stabilize the G4 structure through distinct mechanisms, including direct stacking interactions and effects on loop conformation. At the 3'‐end, G23 remains outside the G‐quartet core and extends from the G4 without direct involvement in G‐quartet assembly. In agreement with this structural arrangement, the 1D ^1^H NMR spectrum of the G23T mutant (Figure S9) displays an imino proton pattern similar to that of *Ch*OGRE23.

Interestingly, C8 interrupts the second G‐tract between G7 and G9 and is extruded from the G‐quartet core. C8 adopts an *anti* glycosidic conformation, as indicated by the absence of a strong intra‐residue H1'‐H6 cross‐peak in the 50 ms NOESY spectrum. In total, 12 C8‐involving NOE‐derived distance restraints were used in the final structure calculation, including 9 intra‐residue NOEs and 3 inter‐residue NOEs with neighbouring G7 protons. The limited number of inter‐residue restraints involving C8 is consistent with an extruded, outward‐facing orientation rather than a conformation stabilized by extensive contacts with adjacent residues. Furthermore, the observed proton chemical shifts of C8 fall within the expected range for cytosine residues, consistent with an unperturbed local environment. Consistent with this, no imino resonance attributable to protonated cytosine was observed under the conditions used.

Hydrogen‐deuterium exchange experiments provided further support for the G‐quartet arrangement. Notably, the imino protons of G2, G9, G17, and G21 remained among the most protected after 2 h of exchange, independently confirming the central G‐quartet formed by these four guanine residues (Figure S13). In addition, the imino protons of G10 and G16 exhibit slow solvent exchange, suggesting local stabilization mediated by stacking interactions between the second lateral loop, C11‐A12‐T13‐C14‐A15, and the upper G1·G10·G16·G20 G‐quartet.

## 4 Discussion

Accumulating evidence indicates that G4s are noncanonical DNA secondary structures capable of forming in cellular environments [44, 45], where they contribute to critical processes such as transcription and DNA replication [46]. Consistent with this view, G‐rich motifs known as OGREs have recently been identified at replication origins, where they are enriched and required for origin activity [18, 19, 20]. To gain structural insights into OGRE‐derived G4s, we selected the oligonucleotide *Ch*OGRE23 for detailed structural analysis as it exhibited well‐resolved and homogeneous NMR spectra. Our results show that *Ch*OGRE23 adopts a monomeric intramolecular (3+1) hybrid G4 featuring a distinctive cytosine bulge arising from the interrupted residue C8 between two G‐quartet layers. The resolved NMR structure further reveals stabilizing loop and G‐quartet stacking contacts, indicating that loop topology is an important determinant of G4 stability.

In 1994, *Tetrahymena* telomeric repeat d[TTGGGG]_2_ was reported to form the first unimolecular three‑layer (3+1) hybrid G4, characterized by three strands oriented in one direction and the fourth in the opposite direction [47]. Since then, numerous DNA sequences have been shown to adopt this topology. To date, 39 unimolecular three‑layer (3+1) hybrid G4 structures formed by 33 distinct DNA sequences have been deposited in the Protein Data Bank (PDB) up to January 16, 2026 (Table S2). These include human telomeric repeats such as d[GGGTTAGGGTTAGGGT] and d[TAGGG(TTAGGG)_3_], together with a variety of sequence variants of telomeric repeats under Na^+^/K^+^ conditions [48, 49, 50, 51, 52, 53]. Beyond telomeric DNA sequences, unimolecular three‑layer (3+1) hybrid G4s have also been identified in promoter regions of *hTERT*, *BCL2*, *VEGFR‐2*, and *PARP1*, suggesting potential regulatory roles in transcription and DNA repair [54, 55, 56, 57]. Notably, five DNA sequences, including a sequence from the HIV‐1 long terminal repeat (LTR) promoter region, have been reported to form unimolecular (3+1) G4s composed of three G‐quartets and a duplex–quadruplex junction [58], constructs derived from the human telomeric sequence [59], the nuclease hypersensitive element III_1_ in the *c‐MYC* promoter [60], and the *PIM1* oncogene, which is associated with the triple‐negative breast cancer [61].

Most reported unimolecular three‑layer (3+1) hybrid G4s are composed of one *syn*·*syn*·*syn*·*anti* G‐quartet and two *anti*·*anti*·*anti*·*syn* G‐quartets, a topology referred to as G‐core II, accounting for 27 of the 39 structures shown in Table S2. In contrast, six structures contain three *anti·anti·anti·syn* G‐quartets, corresponding to G‐core III, including G4s from the promoter region of the *VEGFR‐2* [56] and *WNT1* [62] as well as the first intron of the MAX gene [63]. Unlike previously reported structures, the *Ch*OGRE23 G4 described here adopts a G‐quartet arrangement consisting of one *anti*·*anti*·*syn*·*syn* G‐quartet and two *anti*·*syn*·*anti*·*anti* G‐quartets, corresponding to G‐core I. This G‑core topology has previously been observed only in a limited number of unimolecular G4s, including those from human *chl1* intronic sequence [64], human papillomavirus type 52 [65] and the minisatellite region of *KCNN4* [66]. One exception is the *BCL2* promoter G4, for which the structure has not yet been solved, but which was reported to contain two *syn*·*syn*·*syn*·*anti* G‐quartets and an *anti*·*anti*·*anti*·*syn* G‐quartet [52]. Another notable variant is the *Tetrahymena thermophila* telomeric sequence, d(GTTGGGGTTGGGGGTTGGGGTTGGGG), which forms a four‐layer (3+1) hybrid G4 with a 5' snapback containing one *syn*·*syn*·*syn*·*anti* G‐quartet and three *anti*·*anti*·*anti*·*syn* G‐quartets [67].

With respect to loop organization, the loop arrangement observed in *Ch*OGRE23, two lateral loops and one propeller loop (LLP), represents nearly half of all unimolecular three‑layer (3+1) hybrid G4 structures reported to date (Table S2). However, the combination of G‐core I and the LLP loop arrangement observed in *Ch*OGRE23 has previously been observed for the human *chl1* intronic sequence [64], the human papillomavirus type 52 [65] and the minisatellite region of *KCNN4* [66]. A characteristic of these three structures is the presence of an additional zero‑nt (V‑shaped) loop, with the first 5'‐guanosine adopting a *syn* conformation and being positioned within the central G‐quartet. In contrast, in *Ch*OGRE23 the first 5'‐ guanosine adopts an *anti* conformation and is involved in an outermost G‐quartet, differing from all previously reported unimolecular (3+1) hybrid G4 structures in which the first 5'‐guanosine adopts a *syn* conformation. Consequently, *Ch*OGRE23 represents a previously unobserved glycosidic alignment pattern and loop organization within the (3+1) hybrid G4 fold.

In canonical G4 structures, contiguous G‑tracts are typically connected by loops comprising one or more nucleotides. However, G‑tracts may also be interrupted by non‑guanine residues, giving rise to bulges, which are distinct and intrinsic structural elements that further expand the structural polymorphism of G4s. Importantly, such bulges may serve as recognition motifs for proteins or small molecules. Previous studies have demonstrated that bulges can vary widely in sequence, size, position, number, and folding topology [68]. To date, three unimolecular three‑layer (3+1) hybrid G4s containing bulges have been reported, including those formed by the *VEGFR‐2* and *PARP1* promoters [56, 57], which contain 1‐nt adenine bulges, and HIVpro2 derived from the HIV‐1 promoter, which contains 1‐nt thymine bulge [69] (Table S2). In addition, thymine bulges have been identified in parallel G4 folds within the HIV‑1 LTR promoter, the *KRAS* nuclease hypersensitive element (NHE) region, and the antiproliferative DNA sequence *AGRO100* [70, 71, 72]. While bulges involving cytosine residues have been observed in parallel G4s formed at the 5'‐end of the human telomerase RNA [73, 74], the present work represents, to our knowledge, the first example of a unimolecular three‑layer (3+1) hybrid G4 containing a cytosine bulge derived from a replication origin in chicken. The cytosine bulge introduces a localized distortion of the phosphate backbone and creates an exposed heterocyclic edge, potentially creating a recognition hotspot for replication‑associated proteins.

The growing number of G4 structures containing bulges challenges the canonical G4 consensus motif, G_n_N_m_G_n_N_m_G_n_N_m_G_n_ (n≥3 and 1≤m≤7), and has prompted the development of alternative predictive models for G4 formation [75, 76, 77]. In addition, bulges projecting from the G4 core modify the molecular surface and may generate distinctive binding sites for proteins or small‐molecule ligands, offering opportunities for selective recognition and functionalization in chemical biology and therapeutic applications.

G4‐forming sequences are increasingly recognized as functional non‐B DNA elements at replication origins [12, 19]. In addition to their enrichment in promoters, telomeres and other regulatory regions, G4‐forming motifs are frequently found near or within origins of replication, where they can influence origin specification, origin firing and replisome assembly [78, 79]. Thus, origin‐associated G‐rich elements may act not merely as sequence motifs but as structural elements that provide defined molecular surfaces for protein recognition. This is particularly relevant to the chicken β‐globin origin, where G4‐forming motifs have been shown to influence origin positioning and replication efficiency in vertebrates. However, the biological consequence of G4 formation is context‐dependent. Origin‐associated G4s may facilitate replication initiation, whereas stable G4s that persist on the template strand during elongation can impede fork progression and induce replication stress [78, 80, 81]. Recent mechanistic studies have shown that a leading‐strand G4 can stall the CMG helicase, and that replication through such structures requires coordinated CMG bypass and helicase‐mediated G4 resolution [82]. In Xenopus egg extracts, DHX36 promotes CMG bypass of a leading‐strand G4, whereas FANCJ subsequently resolves the G4 to allow DNA synthesis across the G‐rich motif [80]. Cryo‐EM studies of G4‐stalled CMG further revealed that a folded G4 can lodge in the central channel of the MCM ring and perturb the translocation cycle of the replicative helicase [81].

Our study shows that the high‐resolution structure of *Ch*OGRE23 provides molecular insight into one stable G4 fold accessible to a chicken origin‐derived G‐rich sequence. *Ch*OGRE23 adopts a monomeric three‐layer hybrid (3+1) G4 containing a cytosine bulge and two lateral loops. The C8 bulge protrudes from the G4 core and locally distorts the phosphate backbone, while the loop residues cap the terminal G‐quartets and generate an asymmetric molecular surface. Together with the unusual glycosidic alignment involving the 5'‐terminal G1 in the outer G‐quartet, these features distinguish *Ch*OGRE23 from previously characterized unimolecular (3+1) hybrid G4s. Because many G4‐binding and G4‐resolving proteins recognize not only the G‐quartet core but also loop, groove, bulge, and terminal‐quartet features, the cytosine‐bulged surface of *Ch*OGRE23 may provide a selective recognition platform for replication‐associated proteins or small molecules. We note, however, that *Ch*OGRE23 should be regarded as a minimal, structurally tractable unit derived from the chicken β‐globin origin rather than a complete representation of all conformations adopted by the longer *Chick*OG36 sequence or by the chromosomal origin. The conformational heterogeneity of *Chick*OG36 may itself be biologically relevant, as different G4 conformers could present distinct recognition surfaces or differ in their susceptibility to helicase‐mediated resolution. Future studies using longer constructs, chromatinized templates, and replication‐associated protein‐binding assays will be required to determine whether this cytosine‐bulged G4 topology is formed and recognized in a genomic replication context.

In summary, this study provides a high‑resolution structural characterization of a G4 adopted by the G‐rich replication origin sequence *Ch*OGRE23 and highlights a previously unrecognized (3+1) hybrid topology incorporating a cytosine bulge. These findings shed new light on the structural diversity of replication origin‑associated G4s and their potential functional roles in DNA replication. Although the inherent polymorphism of G4s continues to complicate direct structure–function correlations, detailed structural insights such as those presented here are essential for understanding the biological roles of G4s and for guiding the rational design of G4‑targeting ligands.

## Conflicts of Interest

The authors declare no conflicts of interest.

## Supporting information




**Supporting File 1**: chem71389‐sup‐0001‐SuppMat.pdf
Additional Supporting Information can be found online in the Supporting Information section. The authors have cited additional references within the Supporting Information [18, 24, 83].

## Data Availability

Atomic coordinates and list of chemical shifts have been deposited for *Ch*OGRE23 with the following accession code: PDB ID 9WNM and BMRB ID 36785.
