# Supplementary material for: A Cytosine‑Bulged (3+1) Hybrid G‑Quadruplex Formed by the Chicken DNA Replication Origin
Source: Chemistry. 2026 Jul 15;32(39):e71389. doi: 10.1002/chem.71389 (PMC13715453; doi:10.1002/chem.71389)
Supplement: Supplementary file 1 [file CHEM-32-e71389-s001.pdf]

Supporting information

A Cytosine-Bulged (3+1) Hybrid G-Quadruplex Formed by the Chicken DNA Replication Origin

Yingying You[1], Monica Ching Suen[2], Haitao Miao[2], Yanyan Geng[3], Rahman Ud Din[2], Mu Yuan[2], Qiang Xue[2], Xiaoting Li[2], Naining Xu[2, 4], Zhihong Guo[5], Hon-Ming Lam[6], Kwok-Wai Lo[7], Changdong Liu[2, 4] * and Guang Zhu[2, 4, 8,] *

[1] Department of Otorhinolaryngology Head and Neck Surgery, the Eighth Affiliated Hospital, Sun Yat-sen University, Shenzhen, China

[2] Division of Life Science, The Hong Kong University of Science and Technology, Clear Water Bay, Kowloon, Hong Kong SAR, China

[3] Clinical Research Institute of the First Affiliated Hospital of Xiamen University, Fujian Key Laboratory of Brain Tumors Diagnosis and Precision Treatment, Xiamen Key Laboratory of Brain Center, the First Affiliated Hospital of Xiamen University, School of Medicine, School of Public Health, Xiamen University, Xiamen, Fujian, China

[4] HKUST Shenzhen Research Institute, Hi-Tech Park, Nanshan, Shenzhen, Guangdong, China

[5] Department of Chemistry, The Hong Kong University of Science and Technology, Clear Water Bay, Kowloon, Hong Kong SAR, China

[6] School of Life Sciences and the Centre for Soybean Research of the State Key Laboratory of Agrobiotechnology, The Chinese University of Hong Kong, Shatin, Hong Kong, China.

[7] Department of Anatomical and Cellular Pathology, Prince of Wales Hospital, State Key Laboratory of Translational Oncology, Sir YK Pao Centre for Cancer, The Chinese University of Hong Kong, Hong Kong SAR, China

[8] Institute for Advanced Study and State Key Laboratory of Molecular Neuroscience, The Hong Kong University of Science and Technology, Clear Water Bay, Kowloon, Hong Kong SAR, China

* To whom correspondence should be addressed. Tel: 852-2358-8705; Fax: 852-2358-1552; Email: lcd@ust.hk; gzhu@ust.hk

SUPPLEMENTARY FIGURES

Figure S1 (A) G4 forming sequences from the β-globin replication origin in chicken, including *Chick*OG36 and its truncated derivatives, *Ch*OGRE30, *Ch*OGRE29 and *Ch*OGRE23 [7]. (B) The imino regions of 1D ^{1}H NMR spectra for the DNA sequences shown in (A). (C) Circular dichroism (CD) spectrum of (A) in 70 mM KCl and 20 mM potassium phosphate buffer collected recorded at 25 °C. (C) Comparison of the native PAGE migration of (A) with reference samples, DNA size markers (dT12, dT24 and dT48); 93*del* (d[GGGGTGGGAGGAGGGT]), a dimeric G4; *htel*21T18, (d[(GGGTTA)2GGGTTTGGG]), a unimolecular G4.

A

Name	***Sequence***		
***Chick*OG36**	GGGGGA	GGGAACGCGGCATCAGGGTGGGG	CTCAGGG
***Ch*OGRE30**		GGGAACGCGGCATCAGGGTGGGG	CTCAGGG
***Ch*OGRE29**	GGGGGA	GGGAACGCGGCATCAGGGTGGGG	
***Ch*OGRE23**		GGGAACGCGGCATCAGGGTGGGG	

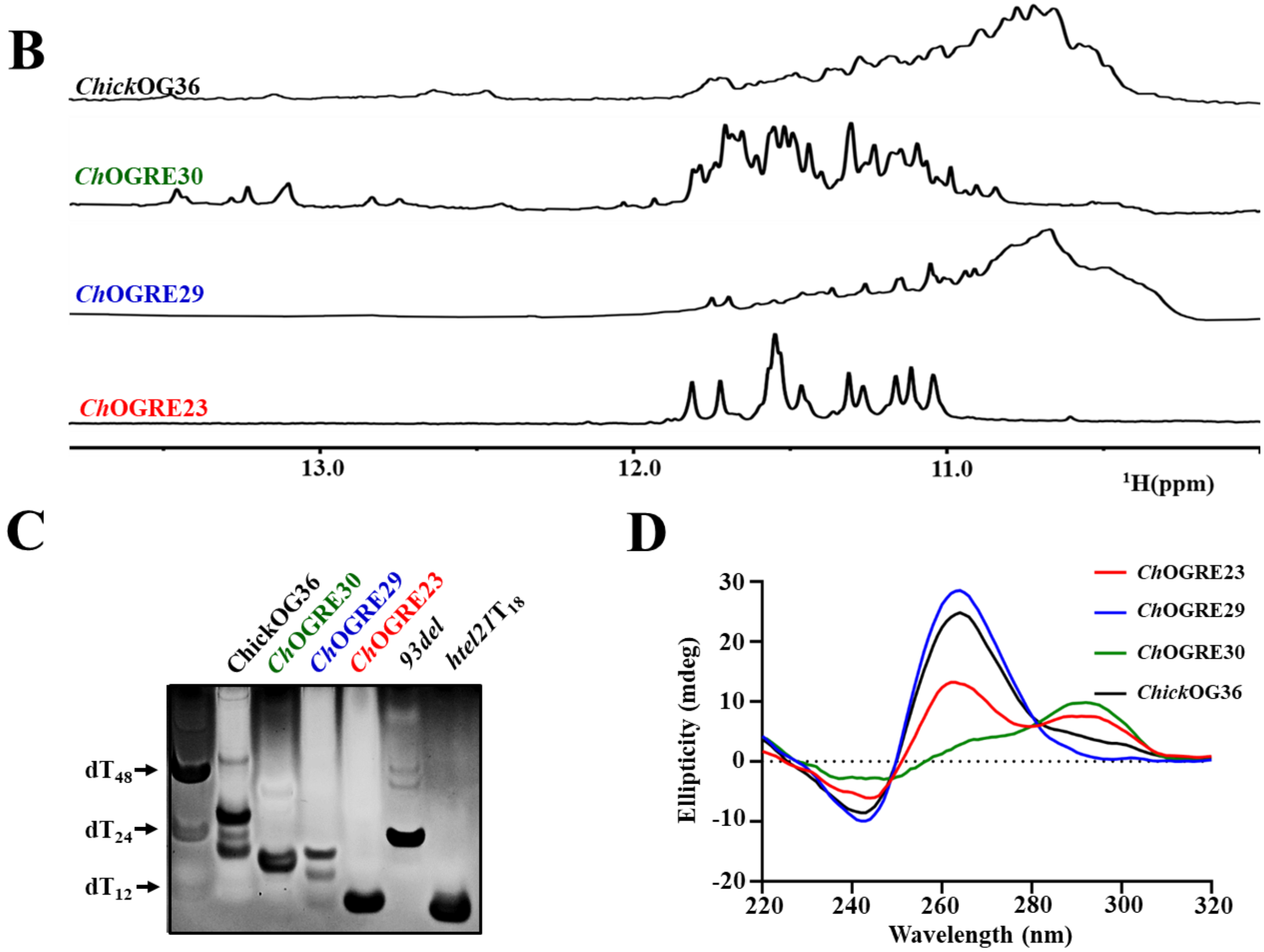

Figure S2 The imino region of 1D ^{1}H-NMR spectra of *Ch*OGRE23 with the assignment of guanine bases in G-quartets being indicated over the reference spectrum on top. The signal of the imino proton of the 3' terminal G23 was not observed indicating G23 is not hydrogen bonded. Guanine imino protons were assigned with 1D ^{15}N-filtered HSQC spectra of samples containing site-specific low-enrichment (5%) ^{15}N-labelled oligonucleotides at the indicated positions. Spectra were recorded at 800 MHz (1D ^{1}H-NMR spectrum and 1D ^{15}N-filtered HSQC spectra) at 25°C in 10% D_2O, 70 mM KCl, 20 mM potassium phosphate buffer (pH 7.0).

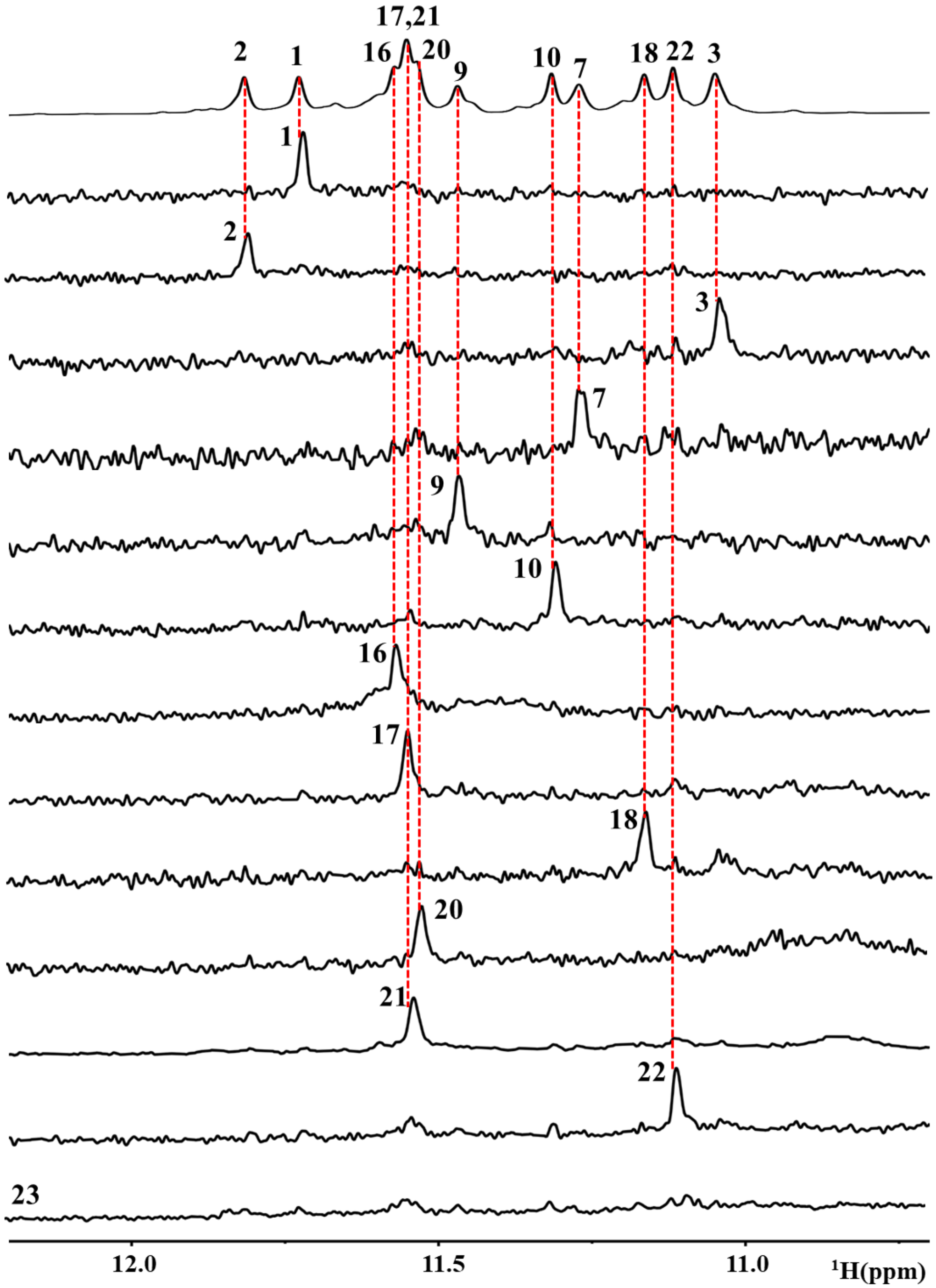

Figure S3 Expanded aromatic C8-H8/C6-H6 region of the 2D ^{13}C-^{1}H HSQC spectrum of *Ch*OGRE23 [48]. The spectrum was acquired at 800 MHz and 25°C in 70 mM KCl, 20 mM potassium phosphate buffer containing 100% D_2O (pH 7.0) at natural isotopic abundance.

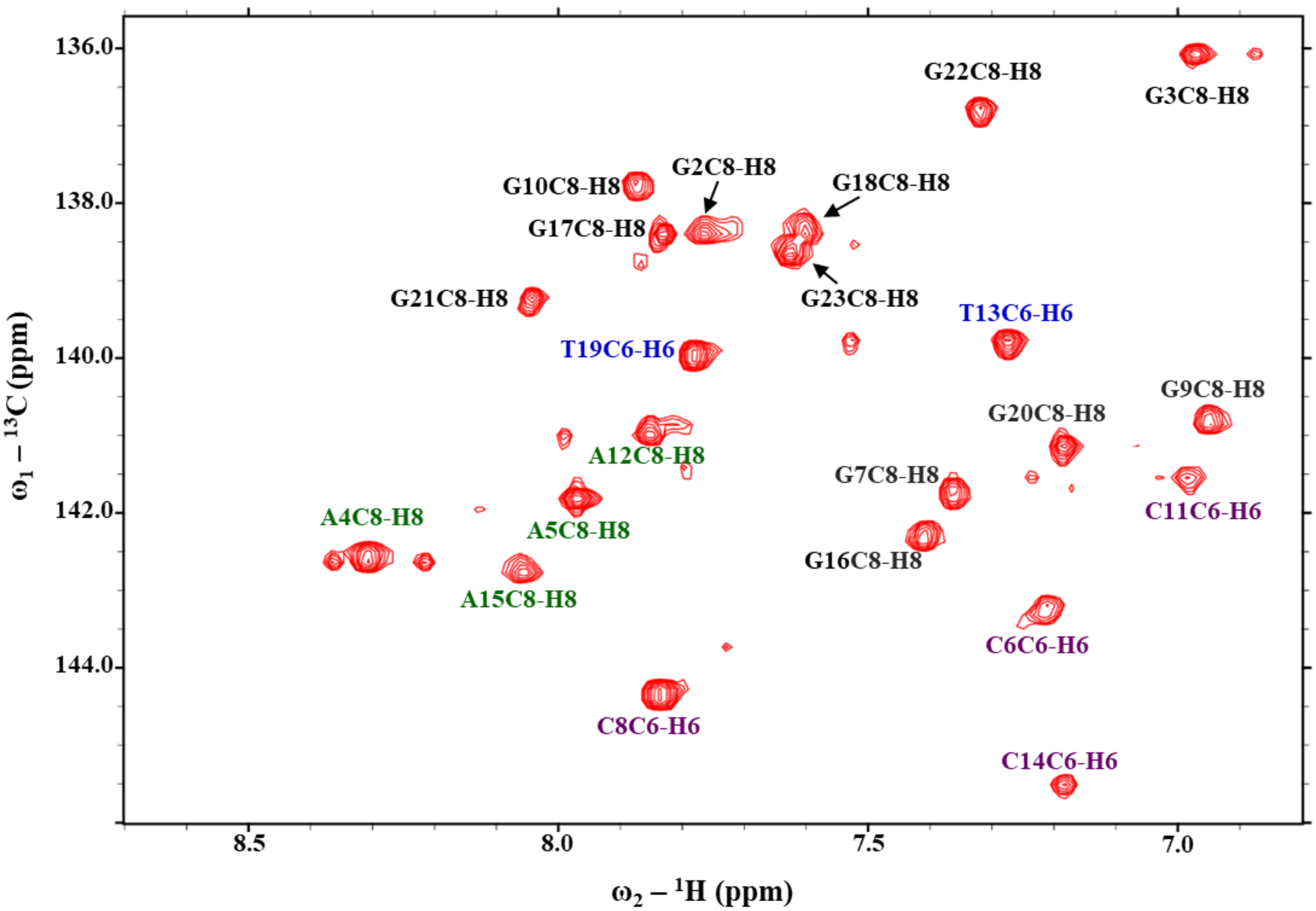

Figure S4 Expanded H1'-H2' region of the 2D ^{1}H-^{1}H COSY spectrum of *Ch*OGRE23. The spectrum was acquired at 500 MHz and 25°C in 70 mM KCl, 20 mM potassium phosphate buffer containing 10% D_2O (pH 7.0) at natural isotopic abundance.

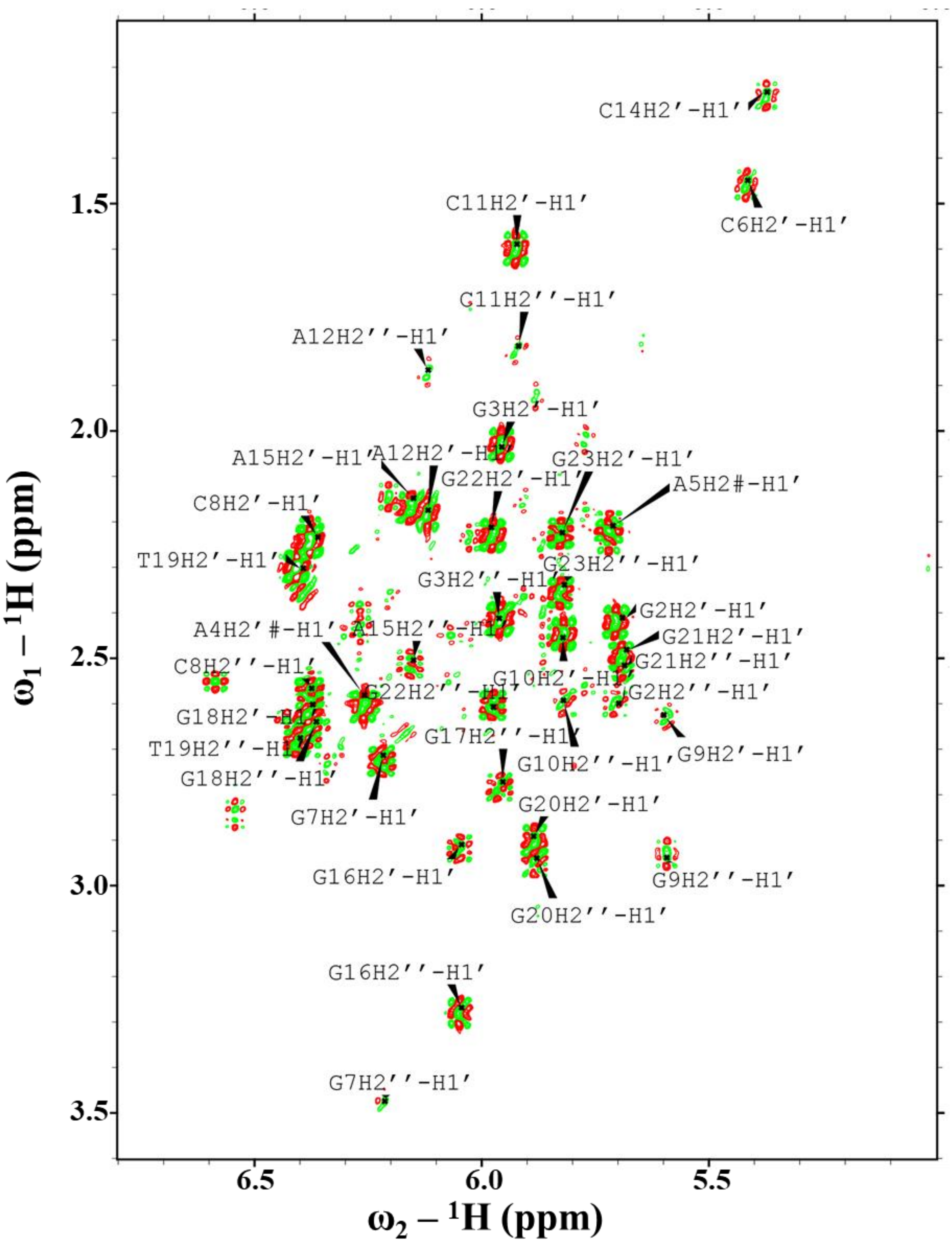

Figure S5 Expanded H1'-H2'/H3' region of the 2D ^{1}H-^{1}H TOCSY spectrum of *Ch*OGRE23. The spectrum was acquired at 500 MHz and 25°C in 70 mM KCl, 20 mM potassium phosphate buffer containing 10% D_2O (pH 7.0) at natural isotopic abundance.

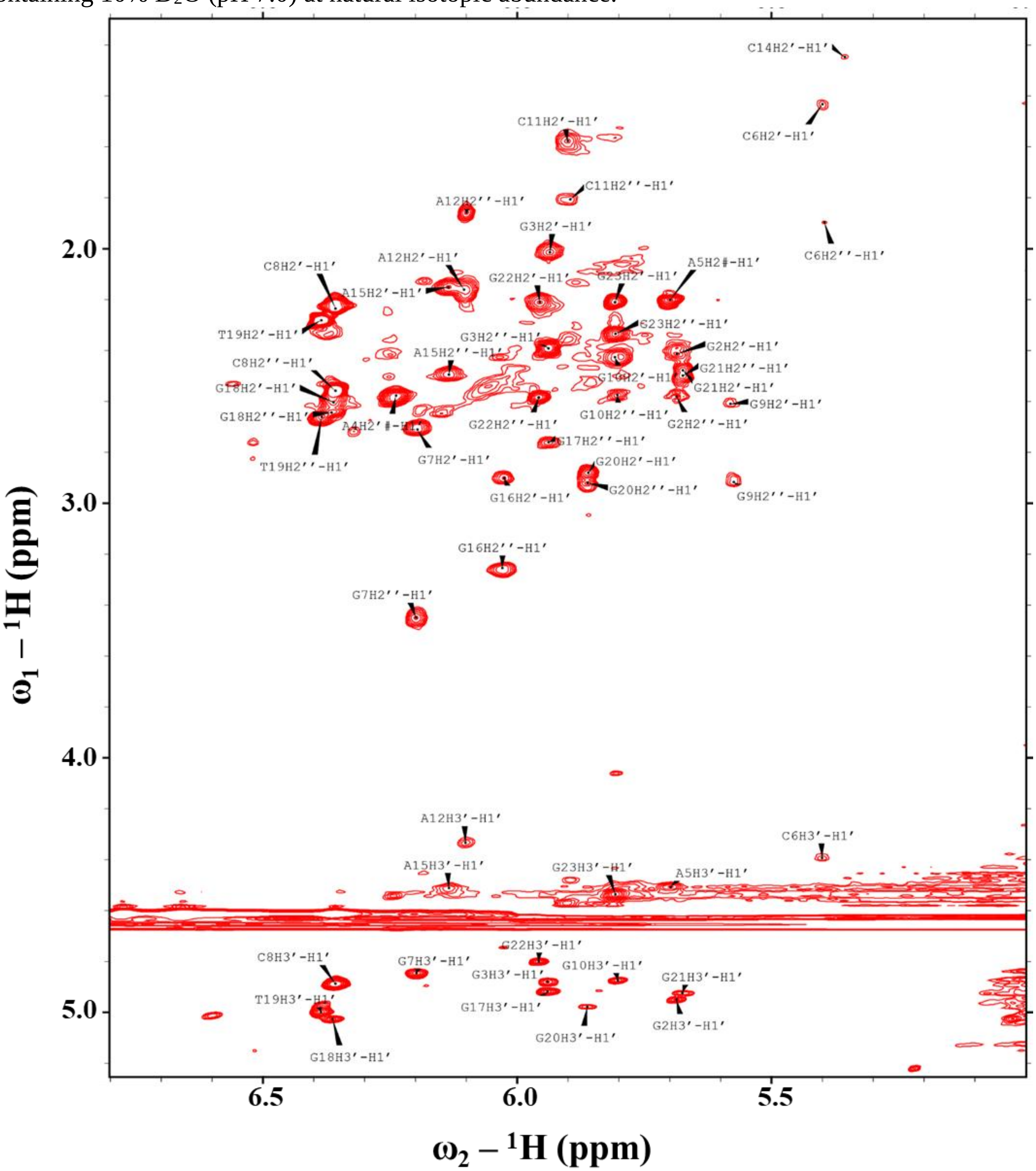

Figure S6 Expanded H5-H6 region of the 2D ^{1}H-^{1}H TOCSY (top) and COSY (bottom) spectra of *Ch*OGRE23, showing cytosine H5–H6 correlations.

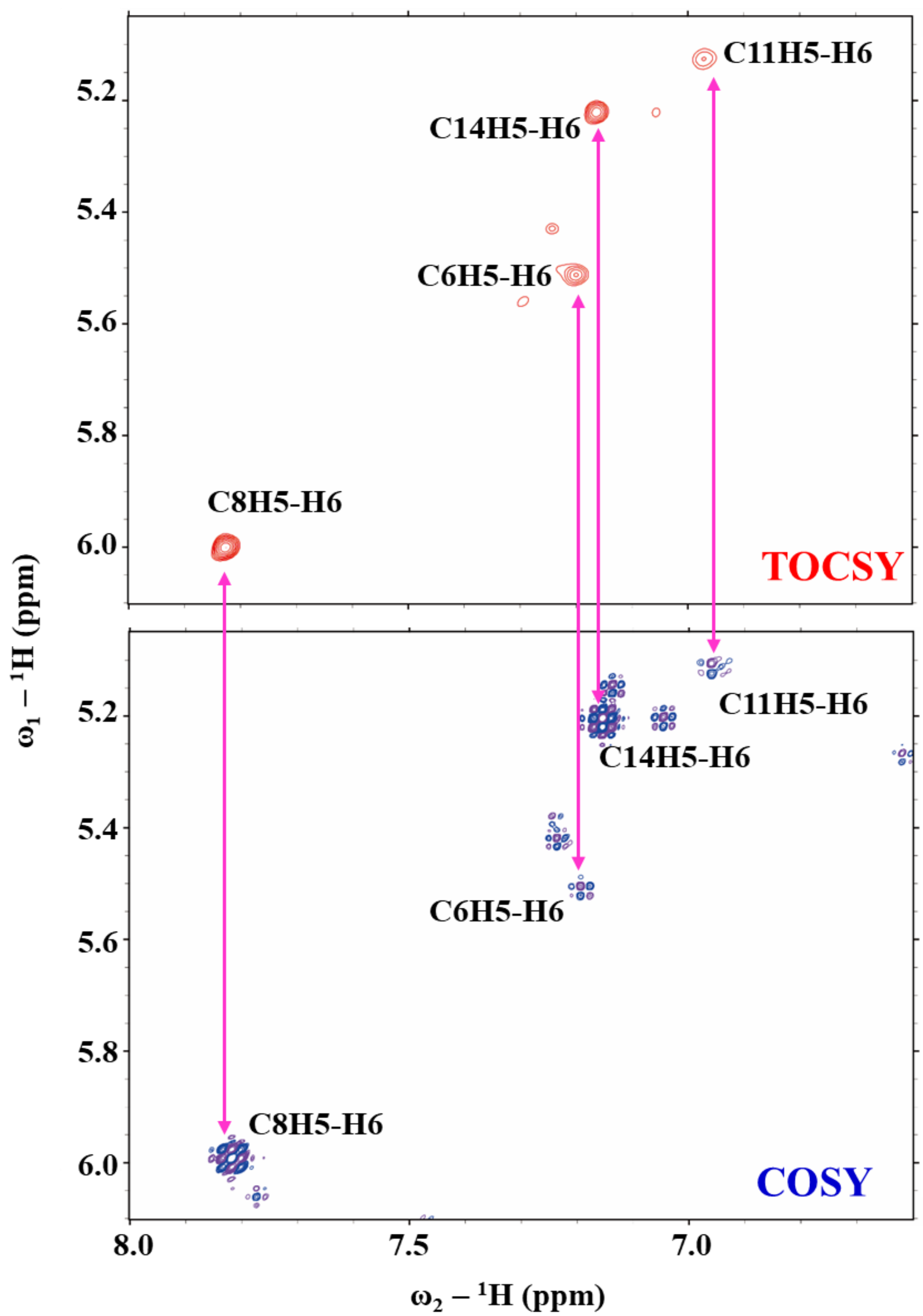

Figure S7 Expanded H5-H6 region of the 2D ^{1}H-^{1}H COSY spectra of *Ch*OGRE23 and the C-to-T substitution mutants C6T, C8T, and C11T. The disappearance of the corresponding H5–H6 cross-peaks in the mutant spectra confirms the assignments of C6, C8, and C11. The dashed boxes indicate the missing cytosine H5-H6 cross-peaks.

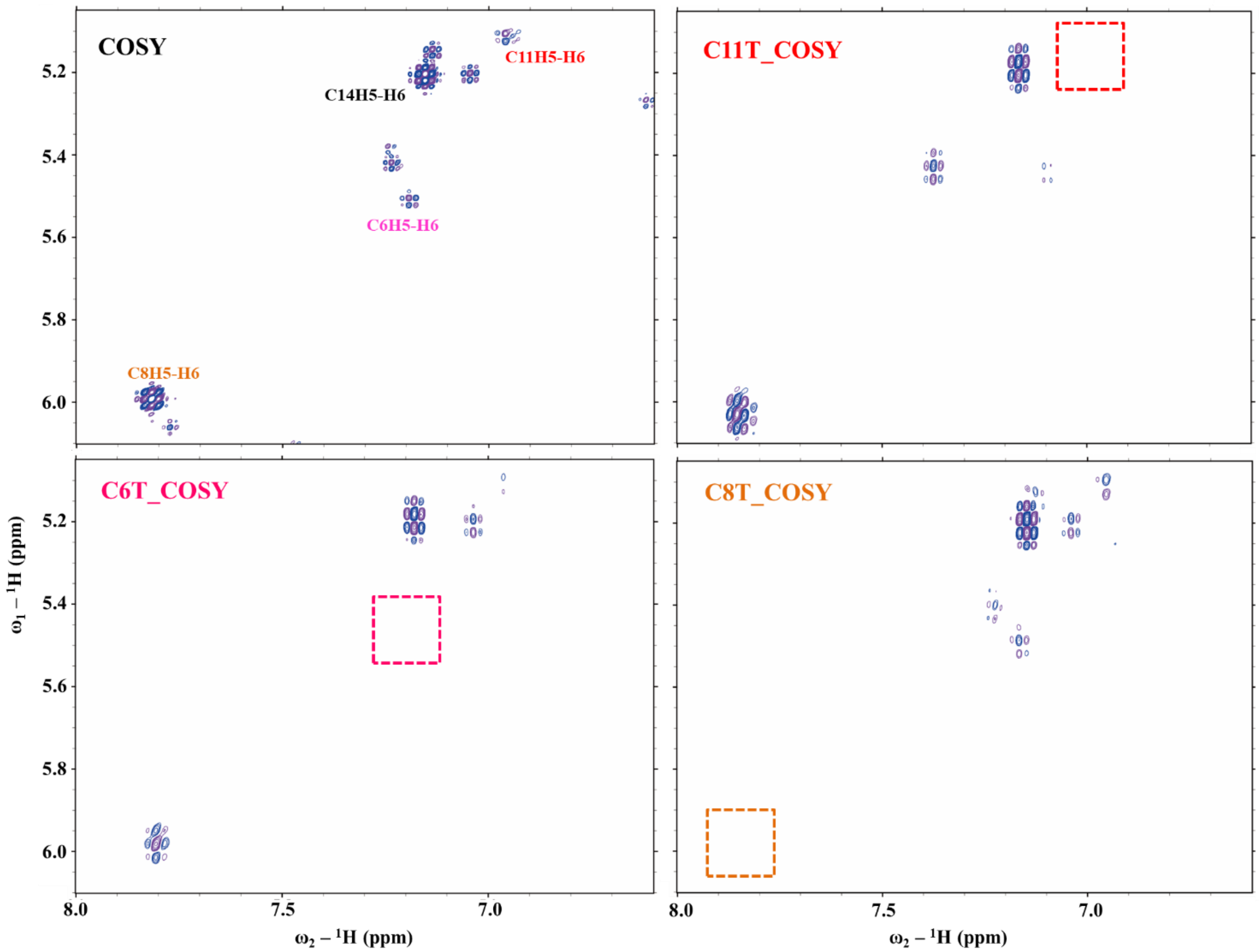

Figure S8 Expanded methyl–H6 (H5#-H6) region of the 2D ^{1}H-^{1}H COSY spectra of *Ch*OGRE23 and the T19C mutant. The disappearance of the T19 methyl-H6 cross-peak in the T19C spectrum confirms the assignment of T19. The dashed box indicates the missing T19 cross-peak.

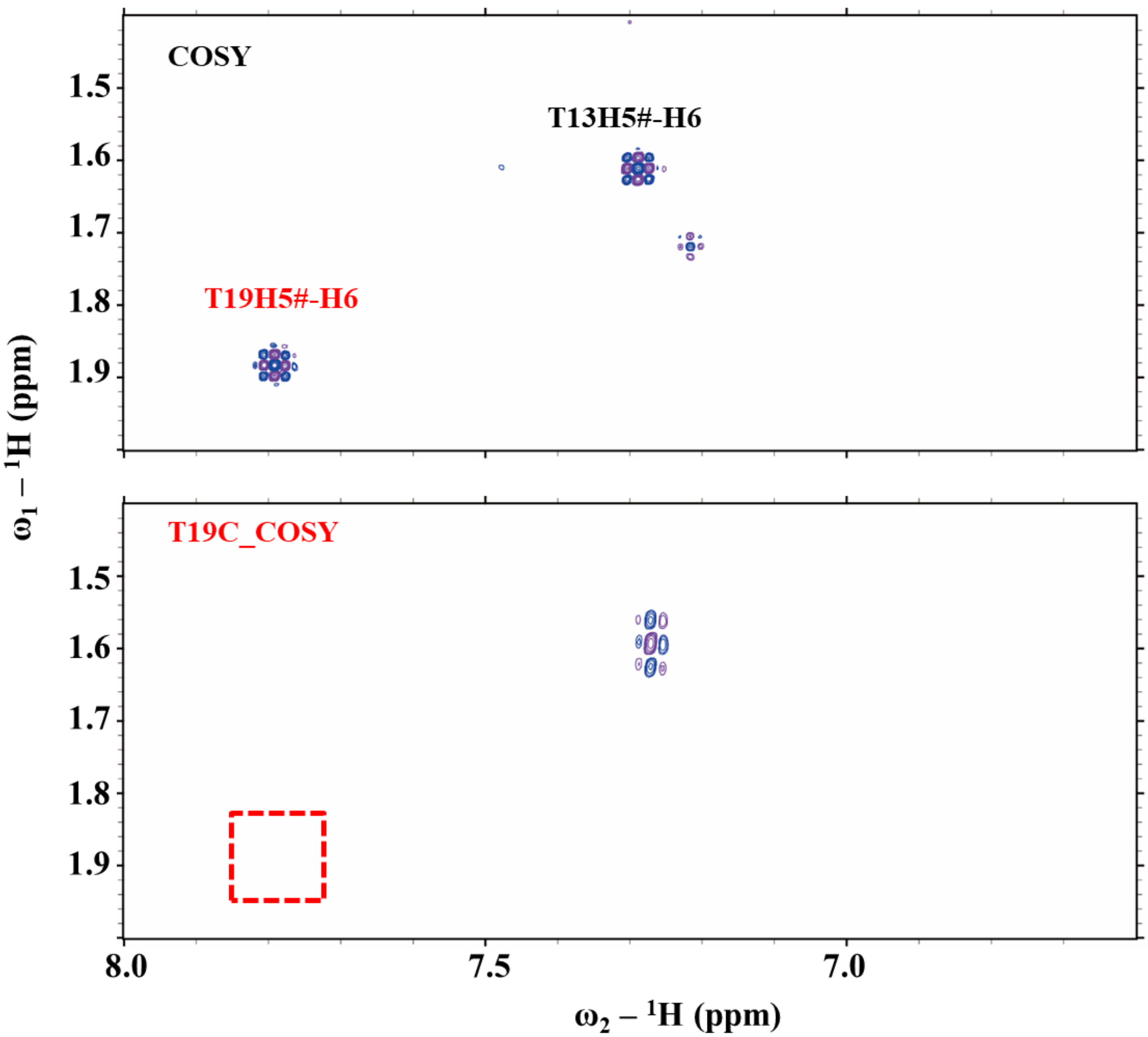

Figure S9 Imino proton region of the 1D ^{1}H NMR spectra of *Ch*OGRE23 and single-base substitution mutants. Spectra of *Ch*OGRE23 and the indicated single-base substitution mutants (A-to-T, T-to-C or C-to-T) are shown. Mutant labels are colored according to the introduced nucleotide: A-to-T substitutions in blue, C-to-T substitutions in red, T-to-C substitutions in orange, and G-to-T substitution in purple.

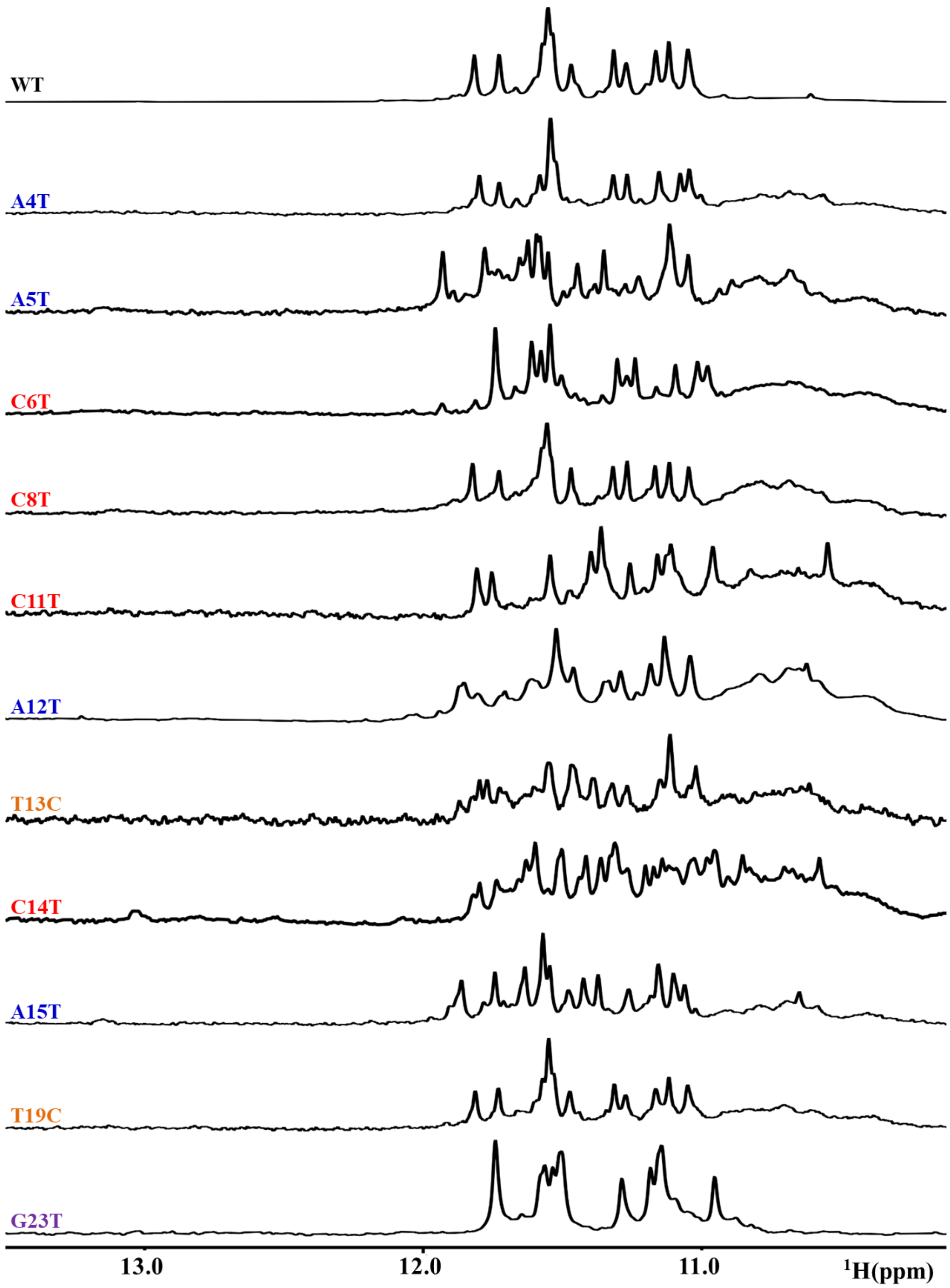

Figure S10 Stacked plot of the short mixing time (50 ms) NOESY spectrum of *Ch*OGRE23. The strong intra-residue guanosine H8-H1' cross-peaks (*syn* glycosidic bonds) are labeled and can be distinguished from weak cross-peaks (*anti* glycosidic bonds).

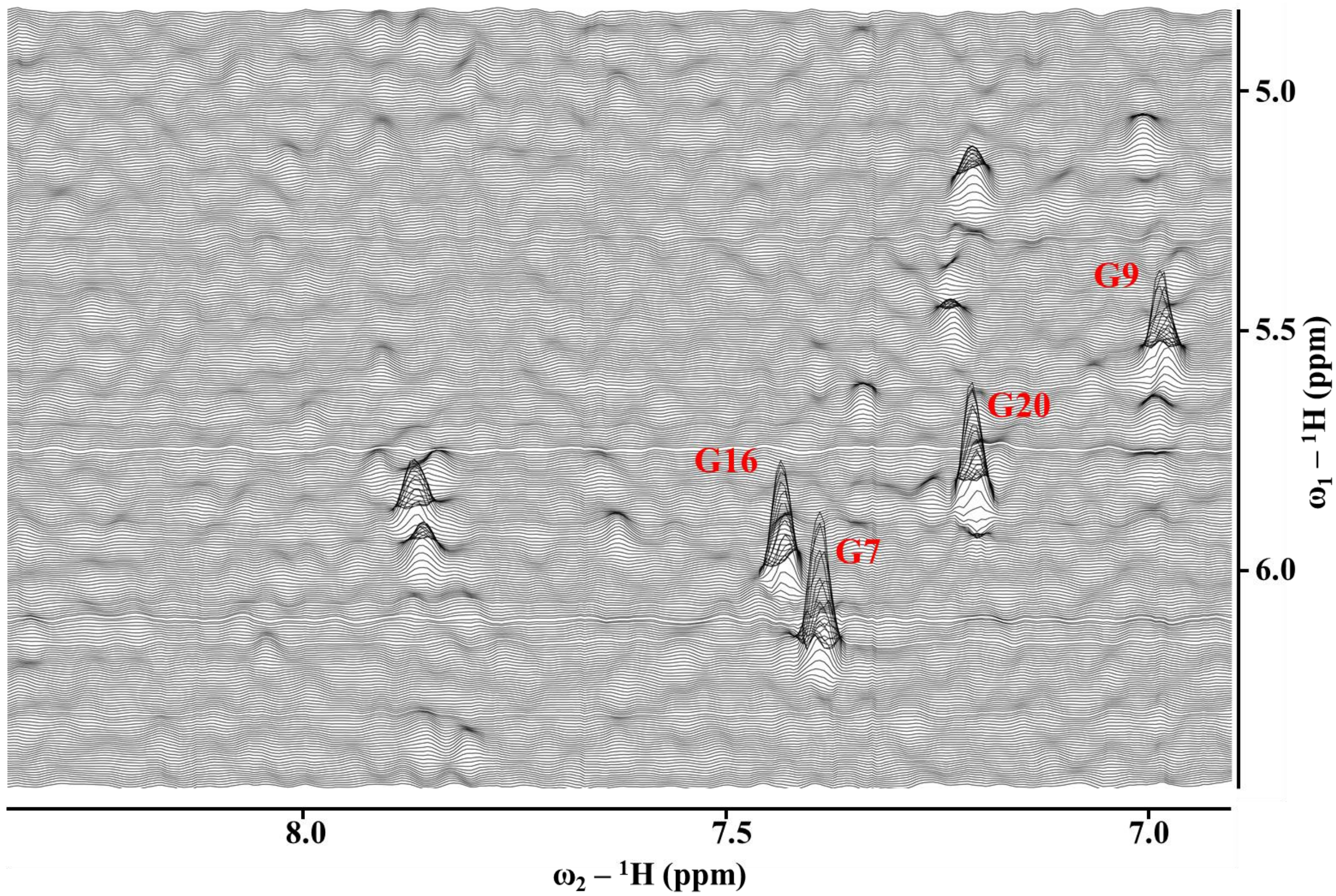

Figure S11 Expanded ^1H-^1H NOESY spectrum (250 ms mixing time) correlating base H8 and sugar H1' protons of *Ch*OGRE23. For *syn-anti* connection, both the *syn*G(i)H1'/*anti*G(i+1)H8 and *syn*G(i)H8/*anti*G(i+1)H1' NOEs are expected to be observed [11b]. The characteristic *syn*G9H8/*anti*G10H1' *syn*G16H8/*anti*G17H1' and *syn*G20H8/*anti*G21H1' NOEs are shown in orange box. The characteristic *syn*G9H1'/*anti*G10H8 *syn*G16H1'/*anti*G17H8 and *syn*G20H1'/*anti*G21H8 NOEs are shown in red box.

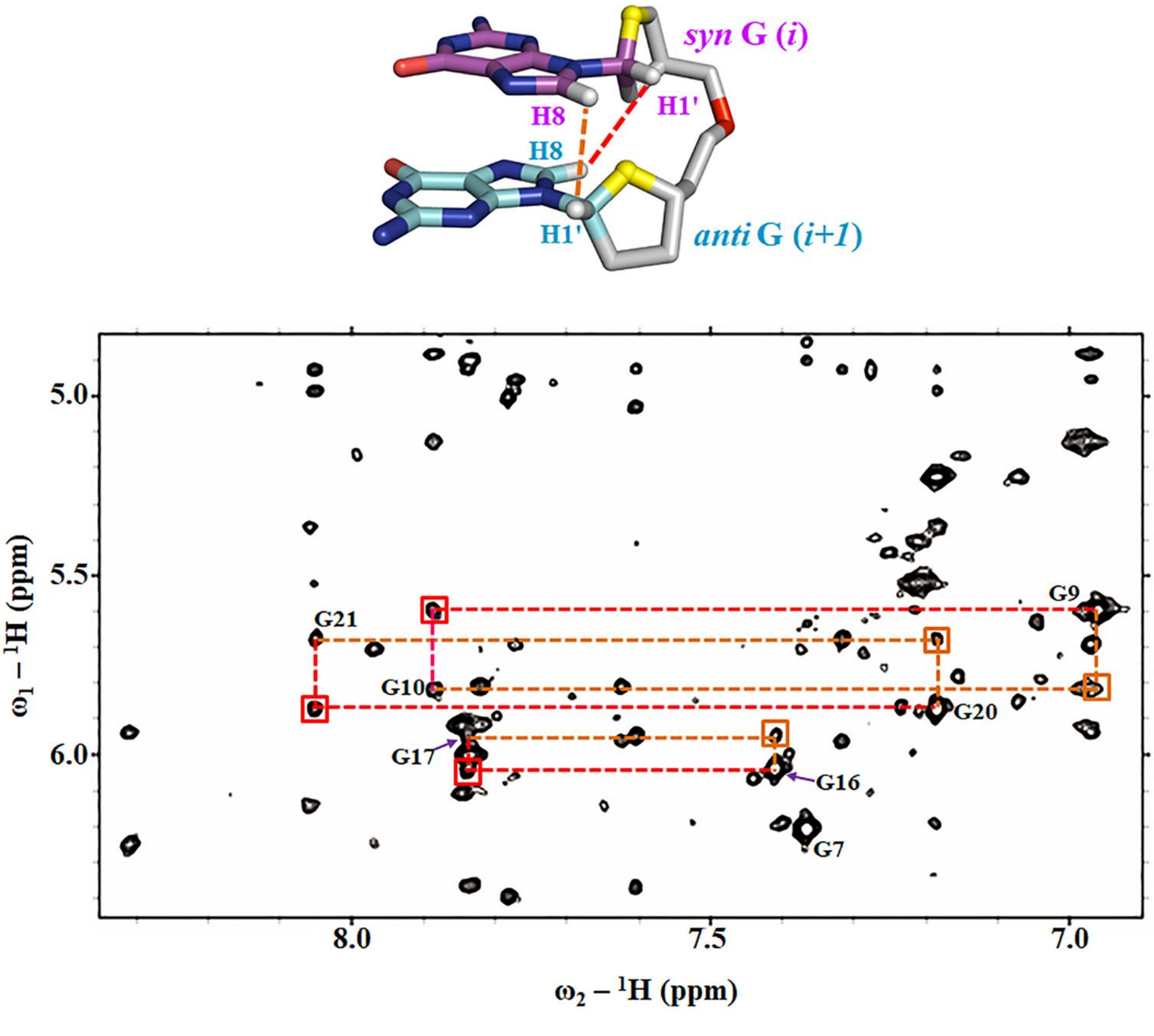

Figure S12 (A) NOESY spectrum of *Ch*OGRE2*3* showing H1-H1 through space correlations labeled as indicated on the spectrum. Medium intensity cross peaks for the guanine pairs (G2/G3, G7/G9, G17/G18 and G21/G22 shown in magenta box) in adjacent tetrads is observed confirming same polarity for the central and bottom tetrad (clockwise) and (G1/G21, G2/G10 and G9/G16 colored by blue box) opposite polarity of the top tetrad (anticlockwise). B. H8-H8 cross-peaks showing sequential connection.

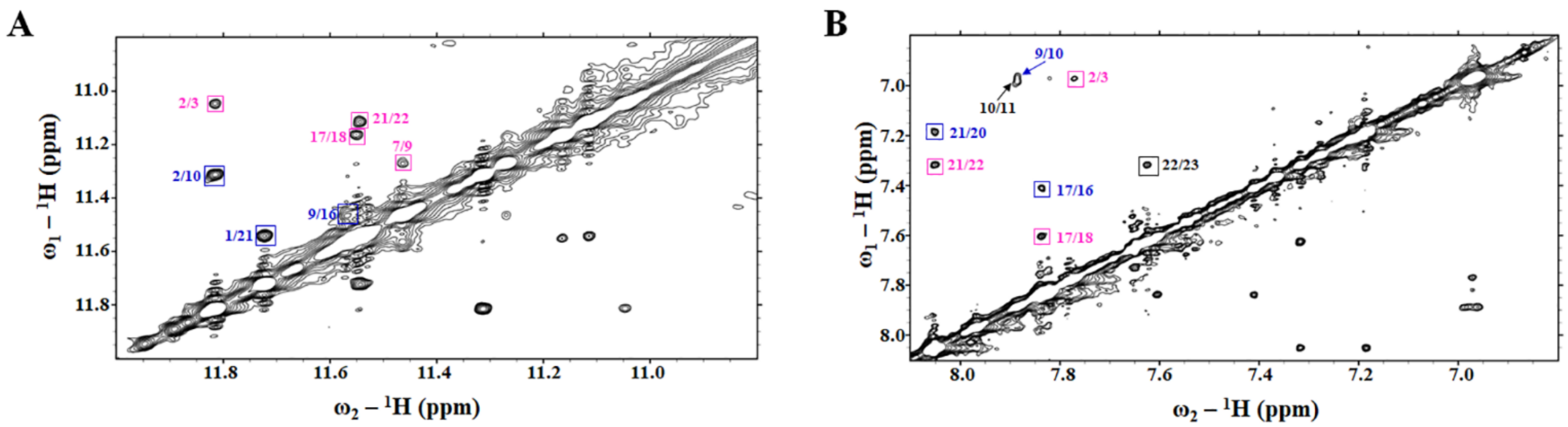

Figure S13. H_2O-D_2O exchange analysis of *Ch*OGRE23. Top: Schematic representation of the hybrid (3+1) G4 adopted by *Ch*OGRE23. Middle: imino proton spectrum of *Ch*OGRE23 acquired in 10% D_2O. Bottom: imino proton spectrum of *Ch*OGRE23 acquired after 2 hours exposure to 100% D_2O.

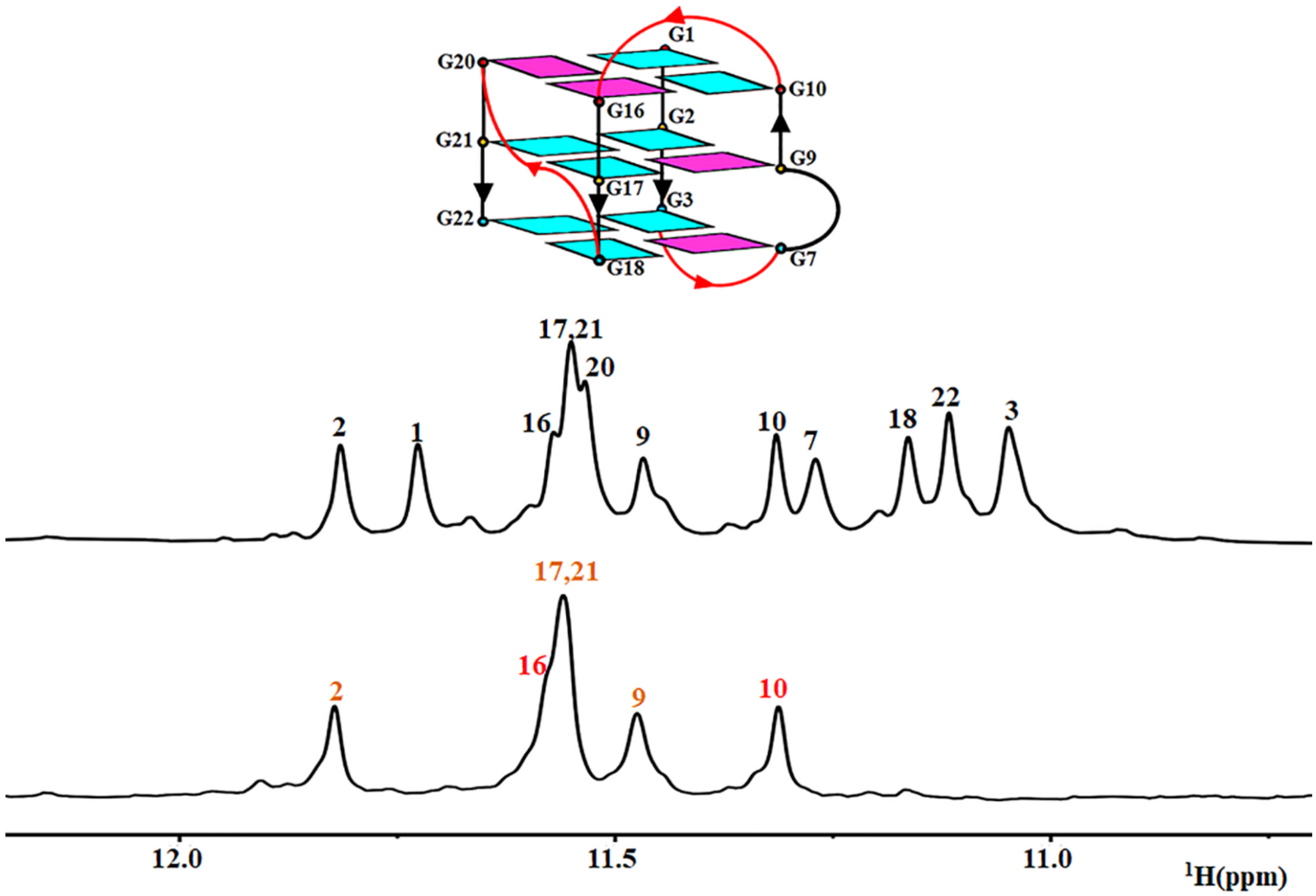

Table S1 Statistics of the calculated structures of *Ch*OGRE23, d[GGGAACGCGGCATCAGGGTGGGG].

NMR distance and dihedral constraints	
Distance Restraints	
Total NOE	***403***
Intraresidue distance restraints	***238***
Sequential (i, i + 1) distance restraints	***82***
Long-range (i, ≥ i + 2) distance restraints	***35***
Hydrogen bond restraints	***48***
Total dihedral angle restraints	***66***
Statistics for structures	
Violations (mean and SD)	
Number of NOE violations > 0.5Å	***0 ± 0***
R.m.s. deviation (Å) from experimental distance restraints	***0.004 ± 0.001***
Number of dihedral angle constraint violations > 5°	***0 ± 0***
R.m.s. deviation (°) from experimental dihedral angle restraints	***0.098 ± 0.001***
Deviations from idealized geometry	
Bond lengths (Å)	***0.002 ± 0.0002***
Bond angles (°)	***0.323 ± 0.015***
Improper (°)	***0.206 ± 0.012***
Pairwise all heavy atom RMSD values (Å)	
Three G-quartets	***0.65 ± 0.11***
All residues	***2.39 ± 0.46***

Table S2. The unimolecular three-layer (3+1) hybrid DNA G4s deposited in the Protein Data Bank (PDB) up to 15 January 2026. The G4s solved in Na$^+$ and K$^+$ solution are colored light gray and light green, respectively. The guanine glycosidic conformations within the G-core are classified into three groups: G-core I, comprising one *syn·syn·anti·anti* G-quartet and two *anti·anti·anti·syn* G-quartets; G-core II, comprising one *syn·syn·syn·anti* G-quartet and two *anti·anti·anti·syn*; and G-core III, comprising three *anti·anti·anti·syn* G-quartets. The loop arrangements of these G4s are classified into four groups: LLP, two lateral loops and one propeller loop; LPP, one lateral loop and two propeller loops; LPD, one lateral loops, one propeller and one diagonal loop; LLPP, two lateral loops and two propeller loops.

The G-core I and the loop arrangement of LLP observed in *ChOGRE23* are highlighted in red.

	PDB ID	Sequence	G-quartet	Loop arrangement	Method	Solution condition
1	186D	d[(T2G4)4]	*G-core II*	LLP	NMR	Na$^+$, pH 6.8
2	7YS7<br>7YS5	d[(G4C)3G4T]	*G-core I*	LPD	NMR	Na$^+$, pH 6.8
3	8KFL	d(G3TAG4CG4CG4CG)	*G-core I*	LPD	NMR	Na$^+$, pH 6.8
4	2E41	d(AG3T2AG3T2AG3T2AG3)	*G-core II*	LLP	NMR	K$^+$, pH 6.3
5	2F8U	d(G3CGCG3AG2A2T2G3CG3)	*G-core II*	LLP	NMR	K$^+$, pH 7.0
6	2GKU	d[T2G3(T2AG3)3A]	*G-core II*	LLP	NMR	K$^+$, pH 7.0
7	2HY9	d[A3G3(T2AG3)3A2)	*G-core II*	LLP	NMR	K$^+$, pH 7.0
8	2JPZ	d[(T2AG3)4T2]	*G-core II*	LLP	NMR	K$^+$, pH 7.0
9	2JSK<br>2JSM	d[TA(G3T2A)4]	*G-core II*	LLP	NMR	K$^+$, pH 7.0
10	2JSL<br>2JSQ	d[TA(G3T2A)4TT]	*G-core II*	LLP	NMR	K$^+$, pH 7.0
11	2KPR	d(G3TG4A2G4TG3T)	*G-core I*	LLP<br>with an additional zero-nt loop	NMR	K$^+$, pH 6.8
12	2KZD	d(AG3IAG4CTG3AG3C)	*G-core II*	LLP	NMR	K$^+$, pH 7.0
13	2LOD	d(G3ATG3ACACAG4ACG3)	*G-core II*	LPD	NMR	K$^+$, pH 6.8
14	5MTA<br>5MTG	d[CAG3T2A2G3(TA)2ACT3AG4T2AG3T2]	*G-core II*	LPD	NMR	K$^+$, pH 7.0
15	5O4D	d[G3TAG3CAG4(AC)2AG3T]	*G-core I*	LLP<br>with an additional zero-nt loop	NMR	K$^+$, pH 6.8
16	5ZEV	d(G3TAC3G3TGAG3TGCG4T)	*G-core III*	LPD<br>with an additional zero-nt loop	NMR	K$^+$, pH 6.8
17	6AC7	d(TG4TC2GAG2CG4CT2G3)	*G-core II*	LLP	NMR	K$^+$, pH 7.0
18	6H1K	d(G3AG2CGTG2C2TG3CG3ACTG4)	*G-core II*	LPD<br>with an additional zero-nt loop	NMR	K$^+$, pH 7.0
19	6L8M	d(G3TCAC2G3CAGTG3CG3)	*G-core III*	LLPP	NMR	K$^+$, pH 7.0
	6L92	d(G3CCAC2G3CAGTG3CG3)	*G-core II*	LPD		
20	6R9L<br>6R9K	d(GCGTG3TCAG3TG3TG3ACGC)	*G-core II*<br>*G-core III*	LPP	NMR	K$^+$, pH 7.0
21	7ALU	d(AG3AG2TGTG2C2TG3CG3)	*G-core II*	LLP	NMR	K$^+$, pH 6.9
22	7ATZ	d(G2TCTGAG3AGAG4CTG3T)	*G-core I*	LLP<br>with an additional zero-nt loop	NMR	K$^+$, pH 7.0
23	7CV3	d(GCG3AG3CGCGC2AGCG4TCG3)	*G-core II*	LLP	NMR	K$^+$, pH 7.0
24	7O1H	d(TGAG3TG3TG3ACGCGCAGCGTG3TA2)	*G-core III*	LPP	NMR	K$^+$, pH 7.0
25	7ZEK	d(G2CTAG3TCAG3TG3TCAG)	*G-core II*	LLPP	NMR	K$^+$, pH 7.0
26	7ZEO	d(G2CTAG3TG3TG3TCAG)	*G-core III*	LLPP	NMR	K$^+$, pH 7.0
27	8KF9	d(G3TAG4CG4CG4CG)	*G-core I*	LPD<br>with an additional zero-nt loop,	NMR	K$^+$, pH 6.8
28	8PSE	d(AG3TAG3CG3CG3TACG3T)	*G-core II*	LPD	NMR	K$^+$, pH 7.0
29	8R4E	d(GCGTG3TCAG3T2G3T2G3ACGC)	*G-core II*	LPP	NMR	K$^+$, pH 7.0
30	8R4W	d(TGAG3TCAG3T2G3T2G3TA2)	*G-core II*	LLP	NMR	K$^+$, pH 7.0
31	8R6D	d[G3(CG)2A2GCAT2(CG)2G3T2AG3TG3]	*G-core II*	LLP	NMR	K$^+$, pH 7.0
32	8S1W	d[G3(CG)2A2GCAT2(CG)2(G3T2A)2G3]	*G-core II*	LPD	NMR	K$^+$, pH 7.0
33	9IMY	d(AG3AG4A2G4TGA2G4A)	*G-core III*	LPD	NMR	K$^+$, pH 7.0
